# The network structure of the urban revolution

**DOI:** 10.1126/sciadv.aee5162

**Published:** 2026-10-14

**Authors:** Giacomo Benati, Sergi Lozano

**Affiliations:** ^1^ Departament d’Història Econòmica, Institucions, Política i Economia Mundial, Universitat de Barcelona, Avinguda Diagonal 690, 08034 Barcelona, Spain.; ^2^ Research Center on Institutions, Politics and Economics (IPERC), Faculty of Economics and Business, Diagonal 690-696, 08034 Barcelona, Spain.; ^3^ UB Institute of Complex Systems (UBICS), Universitat de Barcelona, Martí i Franqués, 108028, Barcelona, Spain.

## Abstract

Long-distance interaction has deep prehistoric roots, but the networks that emerged during the Urban Revolution were unprecedented in scale and organization. How early cities sustained this extensive connectivity in the absence of formal markets or centralized states remains a fundamental question. Focusing on the Uruk Expansion in Mesopotamia (ca. 3900 to 3000 BCE), we assemble the largest spatial dataset currently available and reconstruct transportation networks across three successive phases. Using network analysis, we show that an initially dendritic, river-based system evolved into modular regional clusters and ultimately into a more integrated structure linked by selective long-distance shortcuts. This transformation reduced dependence on key intermediaries and generated small world–like patterns combining strong local cohesion with efficient system-wide reach. These results demonstrate that decentralized interaction processes, supported in part by mobile intermediary communities, played a central role in sustaining early urban connectivity.

## INTRODUCTION

Long-distance interaction is documented throughout prehistory, yet the scale, intensity, and structural organization of connectivity during the fourth millennium BCE—the period commonly termed the Urban Revolution—were unprecedented (*1*). This transition witnessed the rise of the earliest cities, growing demographic concentration, craft specialization, and the emergence of extensive interregional exchange systems (*2*). Despite the central importance of these developments, the mechanisms that sustained long-distance connectivity remain debated, partly because most early urban contexts lack the spatial and chronological resolution required to reconstruct their interaction structures.

The Urban Revolution unfolded across the Near East, but very few regions preserve the evidence required to evaluate how early networks actually functioned. The Uruk Expansion (∼3900 to 3000 BCE) provides an empirical setting to address this question. Often regarded as one of the earliest large-scale episodes of interregional integration (*3*), it combines a dense archaeological record, diagnostic material markers, and identifiable movement corridors at precisely the moment when urban societies were emerging (*4*, *5*). This combination allows reconstruction of interaction networks and, crucially, evaluation of the mechanisms that sustained them (*6*–*9*). Understanding how the Uruk network evolved therefore provides insight not only into a particular historical episode but also into the general processes that underpinned the Urban Revolution more broadly.

Classical accounts interpret the Uruk expansion through centrally coordinated mechanisms, portraying it either as a core-periphery system in which a resource-poor southern core extracted materials from a less developed northern periphery (*10*, *11*) or as a form of state-supported expansion and colonization (*12*). In contrast, subsequent scholarship has emphasized more decentralized and pluralistic processes (*13*–*15*). These include peer-polity interaction among relatively autonomous centers (*16*, *17*), diaspora-led mobility, and broader patterns of demic diffusion (*18*–*21*), each implying lower degrees of hierarchy and political centralization. In parallel, economic perspectives highlight the potential role of decentralized trade systems linking mining regions and agricultural basins through emerging commercial relationships (*22*). Together, these accounts point to a fundamental contrast between centrally coordinated forms of integration and decentralized interaction processes, raising the question of which organizational logic best explains the observed patterns of long-distance connectivity.

However, despite the long history of these debates, there has been no formal, system-level evaluation of how well different relational models account for the evolution of the Uruk interaction system. Recent advances in geospatial analysis and archaeological network science now make such a systematic comparison possible (*23*, *24*). To address this gap, we take advantage of the fact that each competing relational theory carries a distinct logic in terms of the regional organizational structure (Fig. 1), so we can translate them into a set of expected network signatures. (Table 1).

**Fig. 1.**
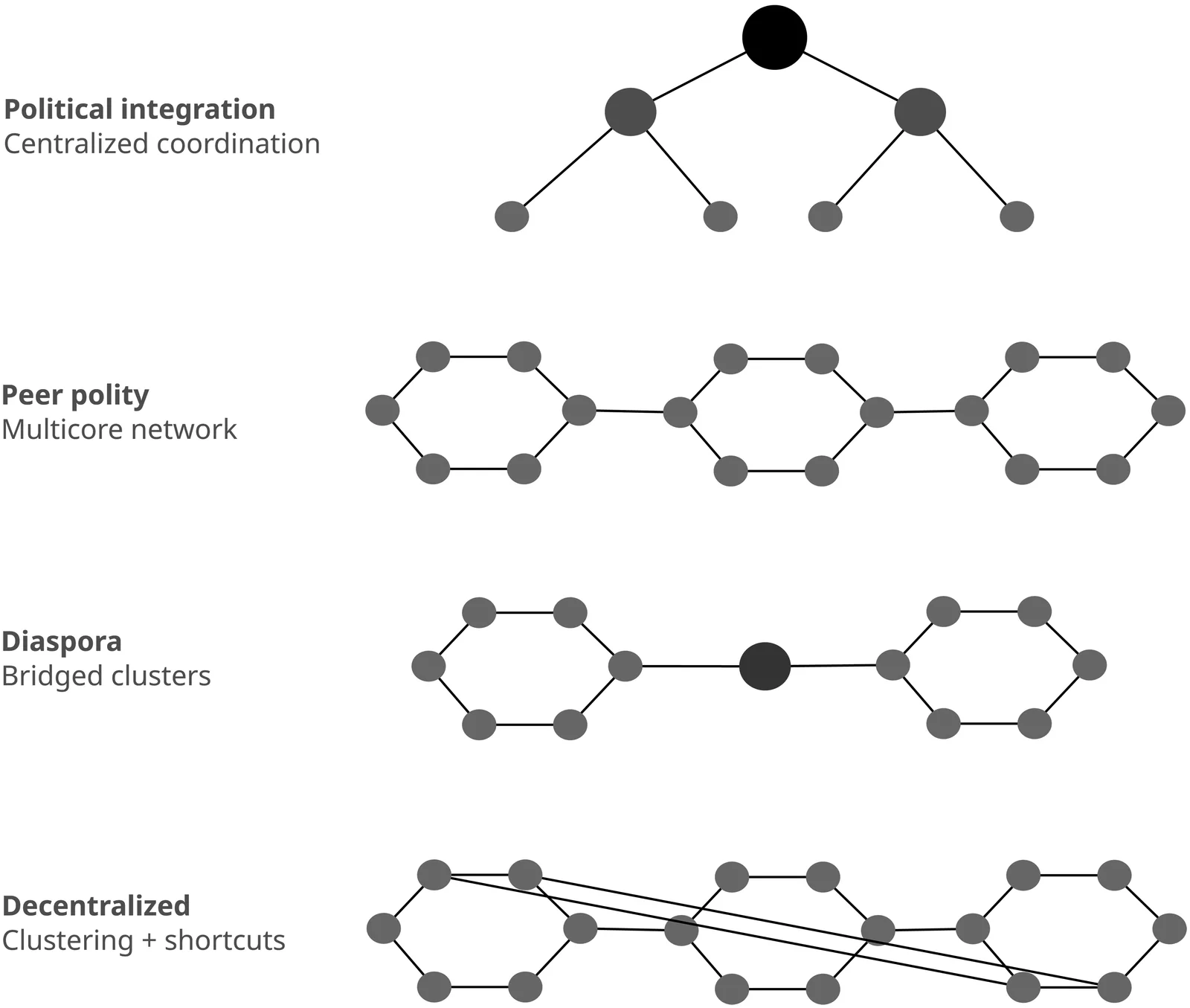
From relational theories to network signatures. Relational theories, predicted network topologies, and observed patterns in the Uruk LC3 to LC5 transportation networks.

**Table 1.** Summary table. Expected network tendencies associated with major interpretations of the Uruk expansion.

Relational theory	Underlying logic	Expected network topology	Structural signature (metrics)
Core-periphery	Extractive core controlling a dependent periphery	Dendritic, hierarchical chains organized along primary corridors	Low clustering coefficient (CC); high average path length (APL); very high betweenness centralization
Political integration	Coordinated infrastructure linking major centers	Reticulated network with persistent hub dominance and direct intercore connections	Moderate clustering; moderate-to-high centralization; relatively short paths between major nodes
Peer-polity interaction	Multiple autonomous centers interacting laterally	Multicore modular structure with regionally bounded clusters	High clustering; multiple intermediate brokers; moderate centralization
Diaspora-driven exchange	Mobile intermediary groups linking regions	Clustered regions connected by a limited number of high-betweenness bridging nodes	Moderate clustering; high betweenness concentration; presence of long-distance bridging links
Decentralized interaction	Locally driven, cost-minimizing link formation	Small world–like structure combining local clustering with selective long-range connections	High clustering; low APL; low centralization

1) Core-periphery (dependency) models are generally associated with dendritic, hierarchical networks with long linear chains, low clustering, and very high centralization.

2) Political-integration models are generally associated with reticulated but centrally coordinated systems, with persistent hub dominance and direct links between major centers.

3) Peer-polity interaction is commonly associated with multi-core modularity, with balanced intermediaries and several regionally significant nodes.

4) Diaspora-driven exchange is often associated with clustered regions bridged by a limited number of high-betweenness enclaves.

5) Decentralized, market-compatible dynamics predict small-world-like networks with high clustering, shorter paths, lower centralization, and selective long-range shortcuts.

Although these expectations are not uniquely diagnostic, they provide a useful framework for comparing the observed network structures with alternative interpretations proposed in the literature.

We apply this framework to the largest geospatial dataset so far assembled for the fourth millennium BCE in Greater Mesopotamia: more than 1700 settlement occupations, curated to 957 reliably dated and geolocated nodes bearing diagnostic elements of the “Uruk cultural package” (*3*). These nodes are paired with communication routes reconstructed independently from historical, geomorphological, and topographic evidence, yielding three time-slice network snapshots (LC3, LC4, and LC5). Our identification strategy builds on a strict separation between the reconstruction of potential connectivity and the observation of interaction. We first define a phase-agnostic system of plausible communication corridors using independent historical, geomorphological, and geoarcheological evidence. We then derive network edges by selecting, from this predefined corridor system, those links consistent with contemporaneous settlement patterns and network connectivity. Because corridors are specified independently of the distribution of Uruk material culture, this approach ensures that network structure is not mechanically driven by archaeological co-occurrence. As a result, variation in network topology reflects the organization of interactions across settlements rather than the procedures used to construct the network itself.

This approach is, however, not devoid of challenges. As a matter of fact, the archaeological evidence in Greater Mesopotamia is uneven due to variable survey coverage, inconsistent recording practices, and differing chronological resolution. To minimize these effects, we standardized all site occupations using the Santa Fe LC framework (*17*), ensuring temporal comparability. Uruk assemblages were coded in binary form, reducing excavation and taphonomic bias while retaining diagnostic markers (*25*). We excluded entries lacking sufficiently reliable spatial or chronological information, removing 23% of observations but preserving more than 90% of securely attested nodes. Still, these excluded entries geographically overlapped considered ones, and therefore, their potential contribution was minimal (fig. S6). Route inference was kept fully independent from cultural-marker distributions, and robustness tests show that LC3 to LC5 structures are resilient to plausible forms of data loss/uncertainty (text S5.2). These steps ensure that the reconstructed networks reflect historical organization rather than documentation artifacts.

The results reveal a clear trajectory: dendritic, river-dominated connectivity in LC3; modular regional clusters in LC4; and integrated networks with selective cross-cluster shortcuts in LC5. LC3 shows some features of a core-periphery configuration, but LC4 and LC5 deviate markedly, exhibiting higher clustering and multiple regional cores (Fig. 2 and Table 2). Relative to geography-aware baselines, LC5 combines dense local cohesion with shorter global paths, producing a small world–like structure. These patterns are difficult to reconcile with strongly centralized dependency or political-integration models, align partially with peer-polity expectations, and show the closest correspondence to diaspora-driven and decentralized interaction dynamics.

**Fig. 2.**
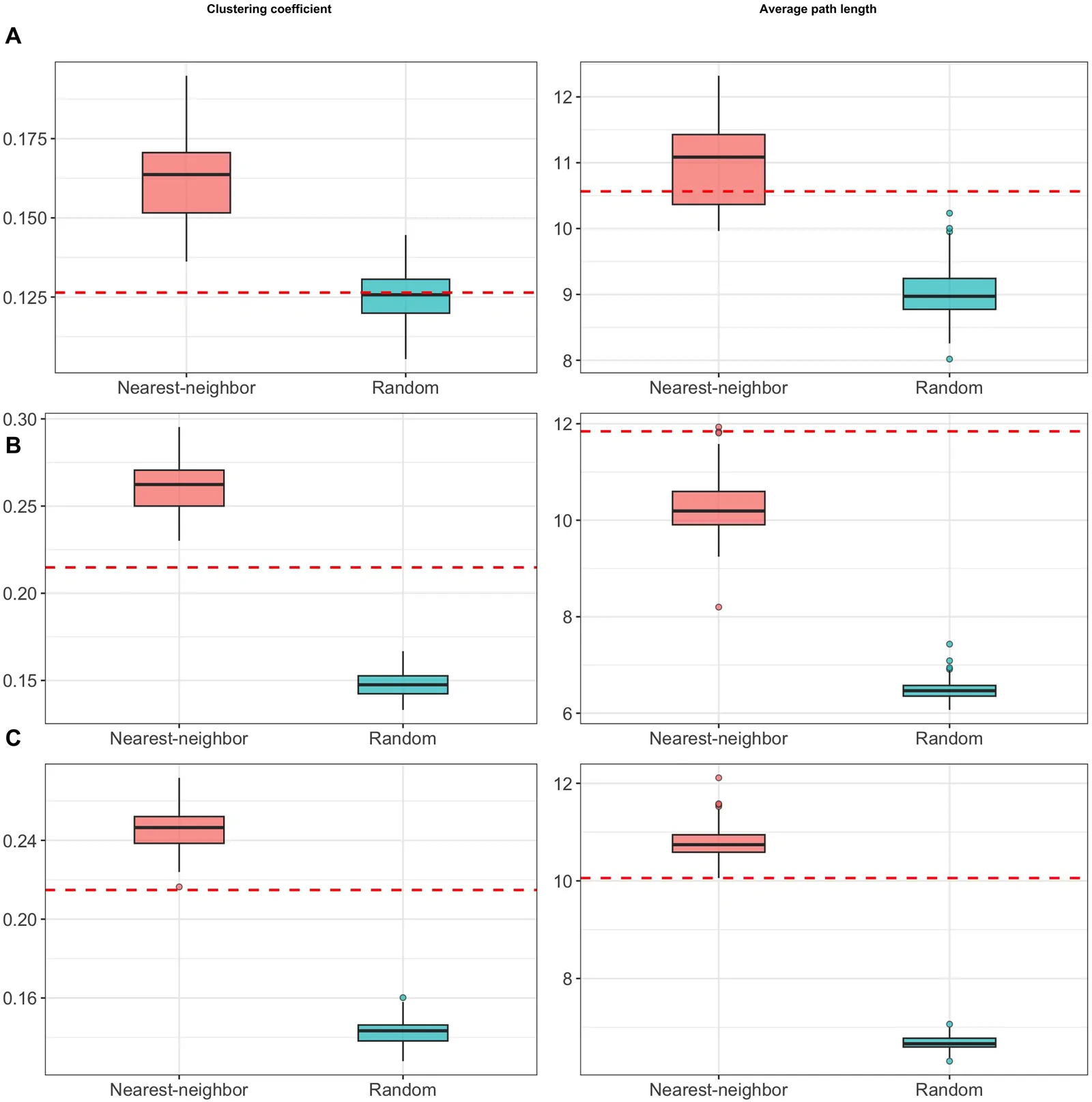
Comparison of empirical and artificial structural characteristics. Clustering coefficient (left column) and Average path length (right column) calculated for the three empirical networks (dashed horizontal lines) and synthetic networks (red and blue boxes). (**A**) LC3; (**B**) LC4; (**C**) LC5.

**Table 2.** Network metrics table. Node size (N), link density (LD), clustering coefficient (CC), average path length (APL), as well as betweenness and degree centralization (Cent_bet_ and Cent_deg_, respectively), for the three empirical snapshots.

	N	LD	CC	APL	Cent_bet_	Cent_deg_
LC3	205	0.014	0.18	10.56	0.41	0.03
LC4	317	0.011	0.30	11.84	0.48	0.05
LC5	380	0.009	0.27	10.06	0.34	0.04

Overall, the findings indicate two key features: (i) Regional integration during the Uruk Expansion appears more consistent with emergent network organization than with strongly centralized forms of control. (ii) Integration is nonmonotonic: Regional interaction systems first intensify locally before becoming more globally integrated through a limited number of intercluster connections.

## RESULTS

### Mapping networks from archaeological evidence

We begin by examining three network “snapshots” representing the organization of connectivity during LC3 (∼3800 to 3600 BCE), LC4 (∼3600 to 3300 BCE), and LC5 (∼3300 to 3000 BCE). Each snapshot consists of settlements occupied during that phase, identified as nodes through the presence of diagnostic Uruk cultural markers and edges derived from reconstructed communication corridors based on independent historical and geoarcheological evidence (*26*). Settlement dates were harmonized using the Santa Fe chronological scheme (*17*).

We define corridors independently from the spatial distribution of Uruk material culture and represent historically plausible routes of movement and communication. The resulting networks (Fig. 3) do not infer links from archaeological co-occurrence but select, from this predefined corridor system, the connections relevant to the interaction system under study based on contemporaneous occupation and network connectivity. This ensures that the network structure is not mechanically driven by the distribution of Uruk material.

**Fig. 3.**
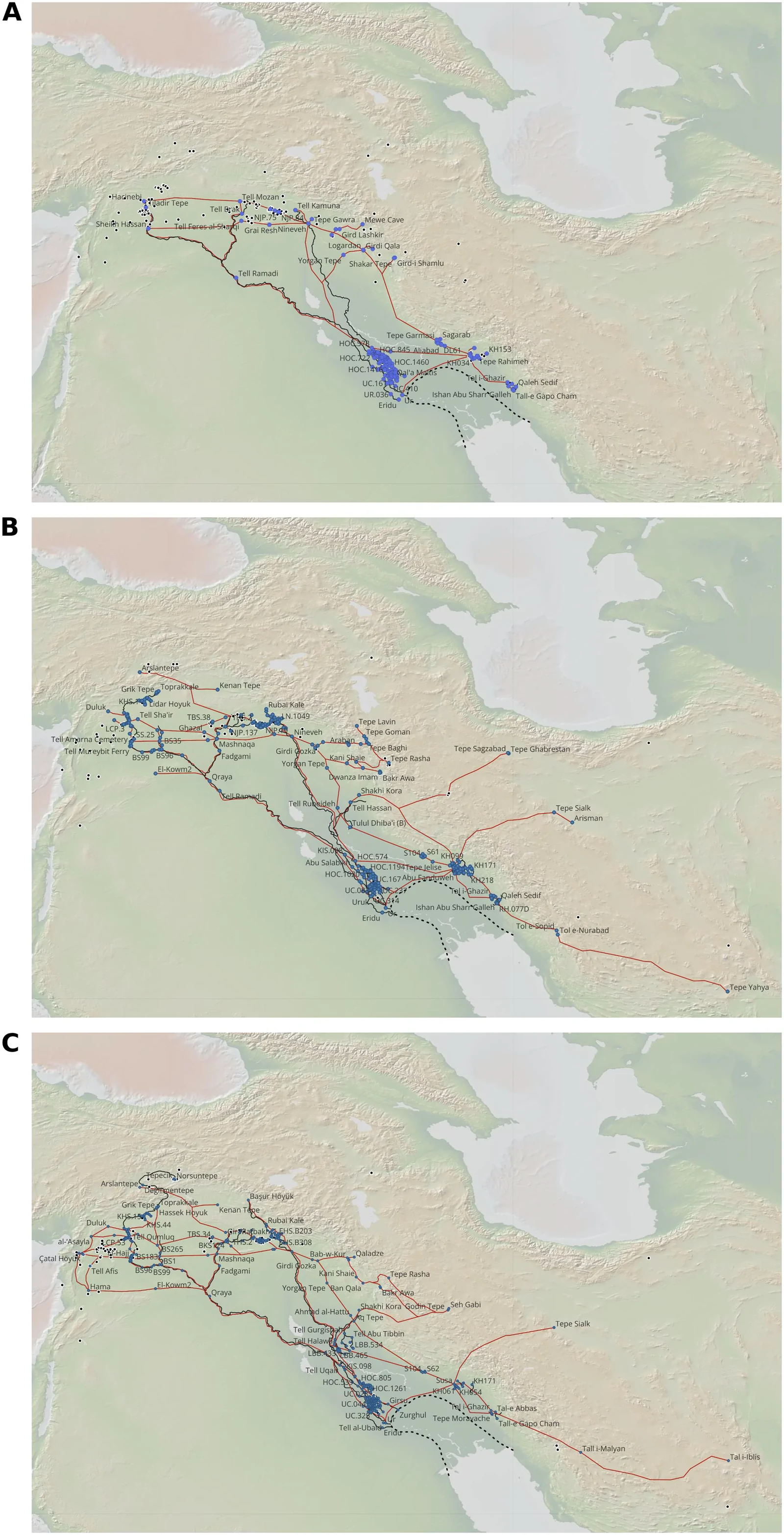
Evolution of the settlement interaction network during the Late Chalcolithic period (LC3 to LC5), showing changes in connectivity in relation to settlements, waterways, and the contemporaneous Gulf shoreline. Evolution of the network across the three time slices [(**A**) = LC3; (**B**) = LC4; (**C**) = LC5]. Blue dots correspond to nodes, small black dots are non-Uruk sites, while red paths correspond to edges. Continuous black lines represent waterways, while the black dotted line represents the Gulf shore in the fourth millennium BCE, based on (*67*). Base map from (*68*) (CC BY 4.0; https://creativecommons.org/licenses/by/4.0/).

To verify that the node selection does not drive the results, we assess the impact of including additional settlements (e.g., non-Uruk sites or sites with less precise chronological attribution). As shown in text S5.2 and fig. S6, these extensions do not significantly alter the observed LC3 → LC4 → LC5 structural trajectory.

Although archaeological data remain uneven across regions (*23*, *24*), most of the sites derive from standardized regional syntheses, and unreliable entries were excluded. Moreover, the presence/absence coding of Uruk assemblages mitigates excavation and taphonomic biases that may affect frequency-based measures (*27*, *28*). Last, robustness tests indicate that the large-scale structural patterns identified here are stable under plausible forms of data loss (*23*, *29*). Together, these steps ensure that the three network snapshots (Fig. 3) provide a credible basis for evaluating changes in connectivity over the Late Chalcolithic. Details on all these robustness tests are provided in Materials and Methods.

### Assessing regional sociospatial organization through network modeling

We use the reconstructed transportation networks to evaluate the degree to which competing relational theories are consistent with the observed network structures, as infrastructure is assumed to reflect the spatial distribution of power and resources among settlements (*30*–*33*). Specifically, drawing on recent modeling literature (*34*–*36*), we applied formal network analysis to examine the structure underlying Uruk’s expansion and its political organization. This involved comparing the structural characteristics of the three network snapshots with the network tendencies associated with the different theoretical perspectives summarized in Table 1. Specifically, such a comparison was based on computational experiments designed to evaluate whether observed connectivity more closely resembled locally cost-minimizing or globally coordinated patterns of link formation.

We began by visually representing the three networks and calculating some basic structural metrics. Figure 3 provides the three network snapshots, where nodes are geopositioned for clarity and links represent either river connections (along the Tigris and Euphrates) or overland routes. Moreover, nodes’ coloring corresponds to the division into structural communities, as determined by the Louvain method (*37*). The first snapshot (LC3) is completely defined by Tigris and Euphrates courses. River links (up to 85.3% of the total) tend to form long chains of nodes mainly connecting North and South Mesopotamia. The second one corresponds to a period of Uruk expansion. While around half of the links are still defined along the two rivers, most of the rest connect to neighboring settlements, thus forming several clusters of nodes. Last, the third snapshot (LC5) shows a retrenchment of Uruk’s expansion around the two rivers and fewer larger modules or clusters (but notice some small ones in Lower Mesopotamia, where links are almost exclusively river based).

These observations are consistent with the structural metrics presented in Table 2 (see also Fig. 4). During Uruk’s expansion, from LC3 to LC5, settlement count grows significantly, while link density remains stable and average clustering increases—a pattern that signals the emergence of the network modules identified above. The evolution of the average path length corroborates this interpretation: The increase from LC3 to LC4 reflects growing local clustering, while the lower value in LC5 indicates the addition of links bridging the underlying clustered structure, i.e., shortcuts. Last, betweenness centralization follows the same trend. The higher values in LC3, and especially LC4, underscore the intermediating role of Lower Mesopotamia in the former period and of sites bridging different clusters in the latter. The subsequent decline from LC4 to LC5 reflects a network with less defined clusters, where bridging sites had lost intermediating power.

**Fig. 4.**
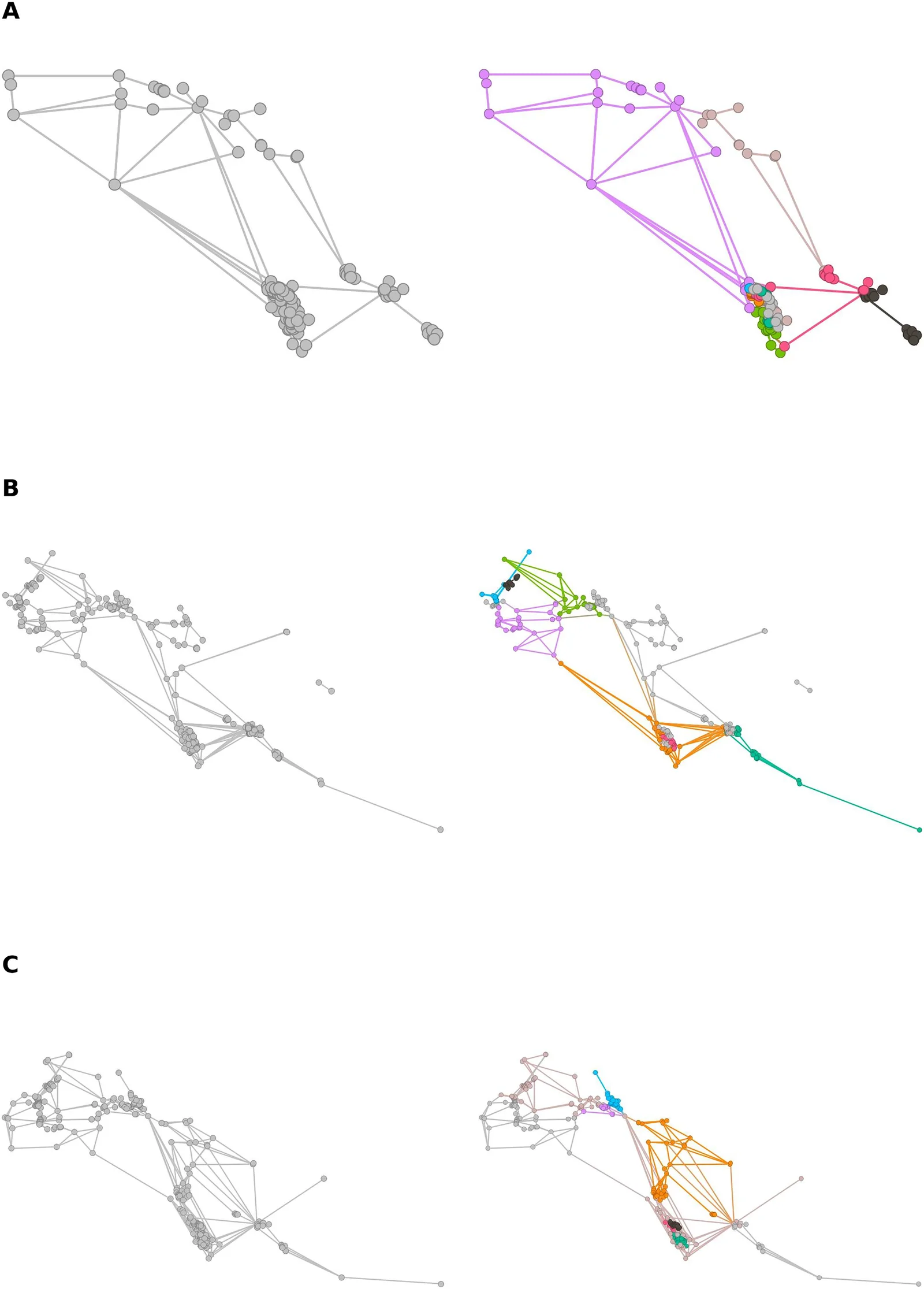
Evolution of the regional settlement network during the Late Chalcolithic, showing changes in connectivity and the configuration of structural communities identified through modularity-based network analysis. Network snapshots corresponding to the three time slices [(**A**) = LC3; (**B**) = LC4; (**C**) = LC5]. Nodes are settlements and edges are defined by routes connecting them. Nodes are arranged according to their geographic position and colored (right column) according to structural community membership as defined by Gephi’s modularity function (with randomization and resolution set to 1.0) (*69*).

These findings offer a preliminary structural characterization of the three snapshots and insights into the transportation network’s role in the studied sociopolitical system. This suggests the beginning of a transition from a river-based corridor to a “small world” pattern, balancing local and global interactions (*38*). However, to formally contrast the network signatures presented in Table 1, we cannot rely only on these results. The range of possible values of any structural metric can reach in a network depends very much on its size (in terms of node and edge counts) among other features. In addition, in our case, the three empirical networks present varying proportions of river versus overland connections. As stated in recent literature, land and river connections played complementary roles in ancient regional transportation systems (*39*). Specifically, in our case study, more overland links provide a greater structural flexibility to reflect different political arrangements. To address these limitations, we compared each empirical network to artificial counterparts of the same size and river link percentage, generated using specific network models to minimize or maximize structural characteristics of interest (i.e., clustering coefficient and average path length; Fig. 2). This allowed us to formally assess each empirical network and compare across the three of them by their position within those upper and lower boundaries. Because the benchmark models isolate purely local or purely global linking processes, the position of each empirical network provides a basis for assessing which classes of interaction processes are more compatible with each phase.

Our two network models are based on the same idea: Rearranging all edges corresponding to land connections while preserving river ones. By holding river structure and spatial constraints constant, differences between empirical and simulated networks are more likely to reflect variation in social and economic organization rather than geographical constraints. The first network model rewires these edges completely at random, while the second one basically draws links among the nearest neighboring nodes (in geographical terms). In both cases, to capture the constraining effect of the geographical space, the distance covered by each new link was kept below the maximum one observed in the corresponding empirical network. By selecting these two models, we considered two extremes where links were established only globally (former case) or only locally (latter one). Each empirical snapshot was compared with 100 synthetic random networks and 100 first-neighbor ones.

The results of our computational experiments are presented in Fig. 2. Comparing empirical network metrics to synthetic models reveals distinct patterns across the three subperiods. The empirical network in LC3 presents a low clustering coefficient and mid to high average path length, in accordance with structure based on settlement chains along river links. In LC4, the values of both metrics are high and fall within the interval corresponding to the first-neighbor model, thus stressing a strong shift to local connectivity. LC5’s values lay between the ranges of the artificial networks. When comparing it to LC4, it also shows relatively high clustering coefficient but a lower average path length, thus reflecting the impact of shortcuts mentioned above. These observations are complemented by the results on betweenness and degree centralization (presented in fig. S5). Both centralization measures present an increasing trend, from low values in LC3—ascribable to the river-based, chain-like connections configuring the network at that period—to high values in LC5—signaling the progressive emergence of regional centers and settlements playing relevant brokerage positions at the extremes of long-distance shortcuts. Last, we also calculated the small-world-ness index (*40*) of our empirical networks. Despite none of the networks reaching the value required to be considered a full-fledged small world, we observe an increasing trend from LC3 to LC5 (see text S5.4).

## DISCUSSION

This study indicates that the observed network trajectory is generally more consistent with decentralized interaction processes than with strongly centralized models. By comparing the observed LC3 to LC5 trajectory (Fig. 3) with the network signatures associated with different theoretical perspectives, we identify a progressive reorganization of interaction structures: from dendritic, river-based chains to modular regional clusters and, ultimately, to a more integrated network incorporating selective long-distance shortcuts. However, network topology alone cannot uniquely identify the social or institutional mechanisms that generated it. Similar structural outcomes may arise through different historical processes, particularly when those processes operate under comparable geographical constraints. Accordingly, the goal of this study is not to identify a single causal mechanism, but rather to assess which broad classes of interpretation are more or less consistent with the observed network structures and their evolution through time.

LC3 displays several features consistent with core-periphery models, including elongated chains and low clustering, indicating strong dependence on a limited set of intermediaries (*10*, *11*, *41*, *42*). LC3 is also partially compatible with political-integration expectations, insofar as both frameworks anticipate centralized movement along major corridors. This interpretation is consistent with archaeological evidence indicating long chains of interaction structured along the Tigris and Euphrates, linking major northern hubs such as Brak and Hamoukar to southern proto-urban centers, where elites controlled the circulation of key goods and access to upstream resources (*13*, *15*, *39*). LC3 is less consistent with peer-polity, diaspora-driven, and decentralized models, all of which imply greater clustering, either through the formation of structural modules or through redundant long-range ties. In this sense, LC3 provides a plausible structural baseline from which subsequent transformations diverge.

By LC4, this structure is replaced by a modular configuration characterized by multiple regional clusters and balanced intermediation, inconsistent with both dependency and strongly centralized political-integration models. In turn, LC4 shows several features commonly associated with peer-polity interaction: Settlements cluster into regional groupings, multiple intermediaries emerge, and no single node dominates intermediation (*43*). This reorganization is also consistent with evidence for the establishment of new enclave sites at key transit points and river crossings, as well as the reoccupation or control of major indigenous centers, indicating a restructuring of interaction around regionally clustered systems connected through strategic links (*39*). At the same time, the appearance of a limited number of longer-range links indicates the beginnings of inter-regional bridging, potentially consistent with diaspora-based or intermediary forms of interaction, although these ties remain relatively sparse at this stage. LC4 therefore can be interpreted as a transition from a corridor-dominated system to a more locally clustered, multicore configuration. More broadly, this pattern is consistent with archaeological interpretations of the Late Chalcolithic as a phase of increasing territorial organization, in which urban centers exerted greater control over their immediate hinterlands while interacting within a broader system of comparable polities (*44*, *45*).

By LC5, the emergence of selective inter-cluster links reduces path lengths while preserving local cohesion, producing a small world–like topology. This configuration is compatible with a system in which connectivity is largely shaped by local interactions and cost-minimizing link formation, rather than by centralized coordination (*46*). Such a structure is difficult to reconcile with either strictly dendritic dependency models or strongly centralized political-integration frameworks. It also goes beyond a purely modular peer-polity configuration, as the presence of intercluster shortcuts substantially reduces overall distances. At the same time, the consolidation of major regional centers and the presence of strategically located enclaves along key corridors—particularly along the Euphrates and at major crossing points—suggests that inter-regional connectivity may have relied on a limited number of intermediary nodes linking otherwise cohesive local systems (*39*, *47*, *48*).

These observations are broadly consistent with a recent archaeological literature arguing that in ancient economies market exchange, redistribution, elite-controlled circulation, gift exchange, and other forms of socially embedded interaction often coexisted and operated at different scales within the same regional system (*49*–*51*). Network evidence is therefore most useful for evaluating the organizational tendencies associated with different exchange systems rather than for identifying specific institutions directly (*49*, *51*).

In this perspective, the LC5 pattern is compatible with diaspora-driven exchange and other decentralized forms of inter-regional interaction. In both cases, most links are established locally—reflecting spatial constraints and interaction costs—while a smaller number of longer-range ties connect otherwise separate regions. These processes can generate networks that are locally dense yet globally integrated, without requiring centralized coordination (*49*, *51*). Archaeological evidence suggests that these patterns may have been supported by a combination of locally embedded interactions and mobile intermediary groups (*52*). In particular, the presence of small diaspora or enclave sites located at key crossing points and along major routes, as well as larger “gateway communities” mediating access to highland resources, provides a plausible historical basis for the emergence of bridging links between otherwise clustered regions (*13*, *16*, *39*, *53*, *54*).

These considerations are especially relevant when viewed against broader archaeological evidence for exchange during the Uruk period. The transition from LC3 to LC5 is broadly contemporaneous with a marked expansion in the geographical distribution of Uruk-related material culture, administrative technologies, and exchange networks across northern Mesopotamia and the Iranian Plateau (*9*, *55*). Recent archeometric research suggests that this growing cultural homogeneity was not primarily the result of large-scale transport of everyday goods. Most ceramics appear to have been produced locally, while administrative devices and recording technologies were generally manufactured and used on local materials (*52*, *56*). Instead, increasing similarity across the Uruk interaction sphere likely involved the circulation of people, technical knowledge, administrative practices, and a more restricted set of strategically important commodities, including bitumen, metals, timber, stone resources, lapis lazuli, and other nonlocal materials (*9*, *52*, *55*). In this perspective, the emergence of regionally clustered systems connected by selective inter-regional links would have facilitated access to geographically dispersed resources while simultaneously accelerating the transmission of innovations, administrative technologies, and material styles, thereby contributing to the growing homogeneity of Uruk-related material culture documented during the late fourth millennium BCE (*9*, *52*, *55*).

More broadly, these patterns find compelling premodern comparanda. Mesoamerican marketplace networks (*57*–*59*)—structured around dense local clusters anchored by periodic markets and linked by selective long-range ties—closely parallel the modularity and shortcut formation observed in LC4-LC5. Similarly, obsidian exchange networks in Mesoamerica and North America illustrate how decentralized, nonstate systems can generate wide-ranging connectivity through overlapping local interactions and sparse long-distance bridging (*60*, *61*). These analogies demonstrate that modular clustering combined with selective shortcuts is not unique to Mesopotamia but recurs in premodern contexts where interaction is not centrally coordinated (*62*, *63*).

Modern transport systems reveal similar structural logics. Early US South rail networks resemble LC3: dendritic, bottlenecked, and export-oriented (*64*). In contrast, the early US North developed modular regional clusters linked by cross-cutting connections, enabling both local cohesion and broader integration—features analogous to LC4-LC5 (*65*). These comparisons suggest that the emergence of selective long-distance links and structural redundancy is a general property of evolving transportation networks, rather than a feature tied to specific institutional forms.

Taken together, the LC3 to LC5 trajectory shows that the expansion of long-distance connectivity during the Uruk period was associated with a progressive reorganization of interaction networks. The shift from river-based corridors to clustered regional structures and, ultimately, to networks incorporating selective long-range shortcuts indicates a move toward forms of connectivity that enhance both robustness and reach. In particular, the addition of intercluster links in LC5 reduces dependence on any single intermediary while maintaining strong local cohesion. This pattern is consistent with interaction systems in which regional connectivity emerged through the cumulative effects of local decisions and intermediary linkages, rather than through centralized coordination (*66*).

In this perspective, the Uruk Expansion may be better understood not as a fully centralized or hierarchical system, but as an evolving network in which regional clustering and selective long-distance connections progressively enabled wider integration. This does not demonstrate the existence of formal markets or unified economic systems. Rather, it points to the emergence of structural conditions—dense local interactions combined with inter-regional bridges—that can support sustained long-distance exchange (*5*, *66*).

This reinterpretation has broader implications for the Urban Revolution. It suggests that the expansion of early cities and the intensification of long-distance interaction may have been driven, in part, by the emergent properties of sociospatial networks, rather than exclusively by top-down political or administrative coordination. In this sense, the Uruk case provides a framework for understanding how large-scale connectivity can arise in early urban contexts through decentralized and spatially embedded processes.

These conclusions should be considered in light of several limitations. Archaeological coverage remains uneven across regions, and undocumented sites may affect local configurations of connectivity. The reconstruction of communication corridors, although based on independent historical and geomorphological evidence, cannot fully capture minor or ephemeral routes. The use of binary coding for Uruk material reduces excavation bias but does not capture variation in intensity or function of interactions. Last, the aggregation of sites into broad chronological phases smooths shorter-term dynamics. While these constraints do not undermine the large-scale patterns identified here, they underscore that the results represent reconstructed network structures consistent with the currently available evidence.

## MATERIALS AND METHODS

### Data acquisition and curation

#### Settlement dataset

Our analysis draws on the largest geospatial dataset compiled to date for the Uruk Expansion phenomenon. We assembled 1748 recorded site occupations from published surveys, excavations, and regional syntheses (see texts S1 and S2 for data sources and compilation and dataset S1 for the full list of settlements and sources). Following systematic curation, we excluded entries lacking sufficiently reliable coordinates or chronological attribution, yielding a final dataset of 957 securely dated and geolocated sites (table S1 and dataset S1). More than 90% of reliably attested nodes were retained. The distribution of curated versus excluded sites, broken down by LC phase, is reported in table S1.

#### Chronological harmonization

To ensure comparability across regions with heterogeneous cultural nomenclature, all local chronological attributions were translated into LC3 (∼3800 to 3600 BCE), LC4 (∼3600 to 3300 BCE), and LC5 (∼3300 to 3000 BCE) using the Santa Fe LC framework (*17*). When available, radiocarbon determinations anchored the placement of occupations within these phases. Chronological assignments for every site, with primary references, are documented in dataset S1.

#### Node definition

Network nodes represent settlements that were occupied during the target LC phase and contain one or more diagnostic elements of the Uruk cultural package, including Uruk ceramics, decorative cones, cylinder seals, tablets or bullae, and monumental tripartite architecture of southern type [(*10*) and text S3]. These markers are widely used proxies for social interaction across the Uruk interaction sphere and preserve well across survey conditions. To reduce sensitivity to excavation intensity, preservation bias, and documentation heterogeneity, all markers were recorded in binary presence/absence form.

#### Reconstruction of edges

Edges represent feasible communication links derived from an independently reconstructed system of historically and geoarcheologically attested corridors (see text S4 for full details). Reconstruction follows three principles

1) Independence from Uruk material distributions, ensuring that edge specification does not derive from the same cultural evidence used to identify nodes.

2) Reliance on long-term corridor persistence, drawing on well-documented continuity of major overland routes across the Syro-Mesopotamian plains from the Bronze Age through the Classical and medieval periods.

3) Geographical plausibility, incorporating physiographic constraints such as river fords, natural passes, paleochannels, floodplain stability, and known topographic bottlenecks.

Operationally, overland routes were digitized from historical maps and published reconstructions of major travel corridors connecting the Syro-Mesopotamian plains, Transtigridian zones, the Zagros piedmont, and routes linking the Middle Euphrates to the Mediterranean basin (extensive discussion and sources are provided in the text S4). Waterways were reconstructed from paleochannel mapping, including avulsion chronologies, aggradation cycles, and Holocene shoreline reconstructions for the Tigris-Euphrates system. These sources define a phase-agnostic system of potential corridors.

From this independently defined corridor system, we activate edges in each LC phase only if two conditions are met: (i) the corridor connects settlements occupied during that phase, and (ii) it contributes to the connected structure of the interaction network. This procedure does not generate links from archaeological evidence but filters a predefined set of feasible routes to retain those relevant for the interaction system under study. As a result, any association between network structure and Uruk expansion arises from the interaction of settlements with a pre-existing corridor system, rather than from the use of archaeological evidence to define connectivity. This design separates the reconstruction of feasible routes from the observation of interaction, allowing network structure to reflect interaction patterns rather than the procedures used to construct links.

#### Robustness and sensitivity tests

The empirical networks constructed following the above-presented procedure would change if new settlements were unearthed, new Uruk materials were identified in formerly non-Uruk sites, or more precise chronologies were reported for sites already included in our database. To evaluate the potential impact of these (and other similar effects related to the archaeological nature of our data), we applied to our networks the robustness test procedure proposed by Brughmans and Peeples (*23*). Basically, these robustness tests consist of progressively removing nodes or edges, recalculating degree and betweenness ranks after each removal, and correlating them with original rankings. In addition to these tests, we analyzed the geographical distribution of sites included in our database but not considered as nodes because they lack a precise-enough chronology (details and results can be found in the text S5 and dataset S2).

### Computational experiments

To properly assess network structure at each phase and being able to compare across empirical networks, we developed a series of computational experiments based on two network models. More concretely, the goal of our experiments was to compare each empirical network with two sets of synthetic ones (i.e., artificially generated using network models) of the same size and density of our snapshots but maximizing globally or locally oriented connectivity.

As mentioned above, our two models rearrange overland connections to create synthetic network equivalent to the empirical ones but exhibiting desired connectivity patterns. The first set of synthetic counterparts was created as a homogeneous random graph (*43*), thus setting connections at random across the whole population of nodes. The second set of synthetic networks was generated by a model establishing links with the closest neighbors in geographical terms [similar to random geometric graphs, studied by Dall and Christensen (*47*)]. When applying this sort of approach, it is usual practice to reshuffle all edges in the empirical network, so that synthetic networks represent ideal null models to identify statistically significant structural signatures. In our case, since our goal was to determine realistic ranges of structural metrics, we limited reshuffling to land connections to mimic real-world constraints (as river connections could hardly be modified by Uruk populations).

For each LC phase, we generated 100 random networks and 100 nearest-neighbor networks. Then, each empirical network was compared to its synthetic counterparts by comparing visually the empirical clustering coefficient and the average path length with the interquartile range of the corresponding synthetic values (All code used for network construction, robustness tests, and computational experiments is provided in data S2).

## Supplementary Materials

### The PDF file includes:

Supplementary Text S1 to S6 Figs. S1 to S6 Tables S1 and S2 Legends for data S1 and S2 References
