# Supplementary material for: The network structure of the urban revolution
Source: Sci Adv. 2026 Oct 14;12(42):eaee5162. doi: 10.1126/sciadv.aee5162 (PMC13665940; doi:10.1126/sciadv.aee5162)
Supplement: Supplementary file 1 [file sciadv.aee5162_sm.pdf]

Science Advances
AAAS

Supplementary Materials for

The network structure of the urban revolution

Giacomo Benati and Sergi Lozano

Corresponding author: Giacomo Benati, giacomo.benati@ub.edu

Sci. Adv. **12**, eaee5162 (2026)
DOI: 10.1126/sciadv.aee5162

The PDF file includes:

Supplementary Text S1 to S6
Figs. S1 to S6
Tables S1 and S2
Legends for data S1 and S2
References

Other Supplementary Material for this manuscript includes the following:

Data S1 and S2

SUPPLEMENTARY TEXT

1. Data Compilation and Sources

The Uruk period corresponds to the Late Chalcolithic 2–5 archaeological phases, dated to between 3900 and 3000 BCE (*9*, *15*, *70*). Following standard chronological conventions (*15*, *48*), we distinguish three phases: LC3 (3800–3600 BCE), LC4 (3600–3300 BCE), and LC5 (3300–3000 BCE).

We assembled 1,748 settlement occupations from published archaeological surveys, excavations, and regional syntheses across Greater Mesopotamia. After removing entries with insufficient chronological or spatial precision, 957 sites were retained. Full counts and breakdowns by LC phase are reported in **Table S1**.

Chronological harmonization follows the Santa Fe LC framework, using radiocarbon determinations where available to refine phase assignments (*70*–*74*). All site-level chronological attributions, including supporting references, are documented in **Data S1**.

For spatial processing and visualization, all georeferenced settlement data and reconstructed corridors were compiled in QGIS (v. 3.36.3), using the EPSG:4326 (WGS84) coordinate reference system.

2. Spatial Data and Geolocation

We built a database by collecting coordinates and settlement data for ancient sites in Greater Mesopotamia occupied between 3900 and 3000 BCE. Following Butterlin's maps (*48*, *75*), we defined the geographical boundaries and collected data from reports, theses, and online resources. We primarily used the *Ancient Near East placemarks* dataset for coordinates (*76*). To standardize dating, we translated local cultural chronologies into three time slices (LC3, LC4, LC5), relying on radiocarbon dates when available.

The bulk of our data came from more recent and standardized projects like the *FloodPlains* (*28*), *Fragile Crescent*, and *CLaSS* (*77*, *78*), which provided reliable information on respectively 3,420 sites from 16 surveys conducted in Iraq, and 1,884 sites from >40 surveys conducted in central Anatolia and Upper Mesopotamia. We manually verified the accuracy of these sources by consulting the primary field reports to integrate as much information as possible. Coordinates and settlement data are listed in **Data S1**.

Entries lacking reliable spatial or chronological information were excluded – e.g. Of the 1,053 identified nodes, only about 9% were excluded during curation, and only 1.8% of precisely dated nodes were removed due to uncertain geographic location. Final inclusion criteria ensure that retained sites form a consistent and comparable spatial dataset across LC phases (**Table S1**).

Although the sample may not be fully representative—a common challenge with archaeological datasets—it is the largest to date on the Uruk phenomenon, far exceeding previous studies (*10*, *48*, *79*).

3. Node Definition (Uruk Cultural Package)

Nodes represent settlements occupied during a given LC phase (LC3–LC5) that exhibit one or more diagnostic elements of the Uruk cultural package. These markers provide widely used archaeological proxies for interregional interaction across the Uruk interaction sphere, independently of the specific mechanisms underlying exchange.

To ensure a consistent and comparable definition of nodes, we do not rely on previously proposed hierarchical classification systems based on site typologies (*10*, *16*, *80*), but instead adopt

an artifact-based approach grounded in observable material evidence. The presence of Uruk material culture—such as distinctive ceramics and administrative technologies—is taken as an indicator of participation in broader interaction networks (*5*, *20*, *81*).

We coded all diagnostic markers in binary form (presence/absence) to reduce sensitivity to excavation intensity, preservation bias, and documentation heterogeneity. Following established archaeological criteria (*10*), we focused on five key elements of the Uruk cultural package:

- Uruk ceramics
- Decorative cones
- Cylinder seals (NB: According to Pittman (*56*, *82*), much of the Uruk administrative practices originated in Khuzistan and then they spread from the southern alluvium proto-urban cores along the piedmont arc during the Middle Uruk period, eventually replacing local stamp-seal based technologies in Upper Mesopotamia. Thus, cylinder seals found outside the southern alluvium, can be considered a strong token of interaction between Upper and Lower Mesopotamia).
- Tablets and clay bullae.
- Monumental tripartite architecture of southern inspiration (NB: We distinguish "standard Uruk" tripartite architecture from the local northern Mesopotamian tradition based on Butterlin´s studies (*48, 83*).

For each site, presence/absence coding and corresponding references are reported in **Data S1**. These durable and widely attested markers provide a consistent basis for identifying nodes across regions and survey conditions, without imposing a priori hierarchical classifications.

4. Corridor Reconstruction (Edges)

We reconstruct connectivity using independently documented travel corridors rather than model-based route inference *(26, 29, 84)*. This choice is motivated by a substantial body of evidence showing that overland routes across the Syro-Mesopotamian plains exhibit strong long-term persistence (*85*). Textual sources from the 2nd millennium BCE indicate that major itineraries already overlapped closely with routes attested in Iron Age, Classical, Medieval, and Early Modern periods (*2*, *10*, *85–88*). This continuity is further supported by the remarkable stability of city-size distributions in Anatolia over several millennia (*89*), and by path-dependent models of ancient route formation (*90*). Crucially, corridor reconstruction is fully independent of the spatial distribution of Uruk material culture. Corridors represent feasible pathways for movement and communication and therefore do not encode inferred flows of material culture. Their subsequent *activation* in a given phase reflects relevance to the interaction system under study rather than derivation from archaeological co-occurrence.

4.1. Overland corridors

We digitized overland routes from published historical maps of long-duration travel corridors. These routes—across the Syro-Mesopotamian plains, Zagros piedmont, and connections to the Mediterranean—show long-term stability across Iron Age, Classical, and medieval periods. We activate corridor segments when they: (i) connect contemporaneous settlements and (ii) contribute to the connected structure of the network.

We reconstructed overland roads based on historical maps of three sub-regions: the Syro-Mesopotamian plains, the Transtigridian corridor, and the Zagros foothills, referencing routes depicted by Algaze (*10*). Additional information on routes, especially those crossing the Iranian highlands and connecting the middle Euphrates to the Mediterranean coast, was sourced from other

scholars (*48*, *75*, *91–93*). The persistence of these routes is attributed to the flat topography and stable positions of rivers and fording points. Corridor activation per phase follows the criteria defined in Section 4.

4.2. Waterways

We reconstructed southern Mesopotamian river systems from paleochannel studies and avulsion histories (Tigris, Euphrates, Diyala). Two major river configurations align with LC3 and LC4–5 phases. Northern waterways were stable and derived from historical floodplain morphology. For the southern Mesopotamian floodplain, we reconstructed water networks using historical maps of river courses based on geoarchaeological and paleoenvironmental evidence. Unlike Northern river Systems, assumed to be stable and derived from floodplain morphology, the Tigris and Euphrates in the south shifted their courses over millennia through a process of aggradation and avulsions, which had a significant impact on settlement patterns and connectivity (*94–96*).

To capture these changes, we relied on:

Allen et al. (*97*)**:** This work reconstructed river avulsions during the 4th millennium BCE by correlating archaeological evidence from Adams (*98*) with paleoclimatic rainfall data. The study uses two cross-sections from around 4000 BCE and 3500 BCE, which correspond to the LC3 and LC4-5 phases of the network.

Jotheri (*94*)**:** For the sake of accuracy, we merged the data from Allen et al. (*97*) with Jotheri's work. Jotheri's reconstruction of ancient river channels is based on a combination of geological, remote sensing, and archaeological data, making it more reliable than reconstructions based solely on-site locations (*99–101*).

Adams (*102*)**:** The course of the ancient Diyala River was mapped using Adams' historical map, which was then cross-referenced with the more accurate site data from the current study (*28*). This analysis showed that the Diyala system developed around the mid-4th millennium BCE, with significant growth in the LC5 period.

Adams (*98*)**, Johnson** (*103*)**, and Alizadeh** (*104*)**:** Smaller channels in the southern plains were reconstructed by observing the overlap between linear arrangements of sites and the paleochannels traced in these previous studies.

Kennett and Kennett (*105*) **and Pournelle** (*67*)**:** The position of the ancient Gulf shoreline was determined by comparing these studies, concluding that it remained relatively stable between 5000 and 3000 BCE.

5. Network Construction and Analysis

5.1 Construction of LC3–LC5 Network Snapshots

We constructed the three empirical networks from edge lists. We construct each network as an edge list linking contemporaneous settlements through activated corridor segments. In our analysis, we combined the visual inspection of the three empirical networks with their characterization using four different network metrics (see **Table 1 in the main text**):

- Number of nodes (**N**), which allows to track the strong growth of the network along the period of study.
- Link density (**LD**), calculated as the number of links present in the network over the maximum possible number of links in it (i.e. if all nodes were connected among them). In

our case, this measure remains stable, meaning that the number of connections in the region also increased steadily throughout the three phases.

- Average Clustering Coefficient (**CC**), measuring the density of triads (triplets of connected nodes), complements the previous measure. Its increase from LC3 to LC4 and LC5 suggests the formation of network clusters (i.e. groups of densely connected nodes), as is observed in Figure 2 of the main text.
- Average Path Length (**APL**), which is the average distance among all node pairs in the network (not in geographical/spatial terms but measured in term of the number of other nodes that have to be transverse). Higher values in LC3 are related to chain-like branches of the network, typical of river routes. As already indicated in the main text, high values of CC and low values of APL (as presented by the LC4 network) is interpreted as a signature of the 'small world' structural pattern.
- Betweenness and Degree centralization (**$Cent_{bet}$ and Deg_{bet}**). Like other centralization measures, this one quantifies how unequally distributed are betweenness (degree) centrality scores in a given network. For betweenness, higher values correspond to scenarios where few nodes concentrate the intermediation power. On the contrary, lower ones indicate that there are several alternative paths connecting each pairs of nodes, so homogenizing betweenness scores across the network. Degree centralization allows to detect the presence of hubs in a network. In our particular case, high degree centralization would be related to the emergence of regional hubs within different clusters.

5.2. Robustness to Missing Data

Archaeological data are often incomplete or subject to preservation and sampling biases (*29*, *106*), which may affect network structure when nodes or links are missing. To evaluate the impact of such uncertainty, we follow the robustness procedure proposed by Brughmans and Peeples (*23*), which consists of progressively removing nodes or edges and assessing the resulting structural changes.

Specifically, we rank nodes by degree and betweenness centrality before and after each removal, and we use the correlation between rankings to measure structural stability. **Figs. S1–S4** present the results of node and edge removal tests for the three empirical networks. In all cases, the networks remain robust to perturbations, although effects are stronger for betweenness centrality, as expected given its sensitivity to global network structure.

To assess the potential impact of including currently excluded sites (e.g., non-Uruk or imprecisely dated sites), we compare their spatial distribution with that of the selected nodes. The two distributions largely overlap, with only minor peripheral differences (**Fig. 1**; **Fig. S6**). This suggests that incorporating additional sites would lead primarily to local adjustments rather than substantial changes in overall network structure.

5.3. Computational experiments

As discussed in the main text, the three empirical networks are not directly comparable due to differences in size and in the share of river-based links. To assess network structure consistently across phases, we compare each empirical network with synthetic counterparts generated under two benchmark models.

For each time slice, we construct:

- a homogeneous **random network** (*43*), where links are assigned uniformly at random, and

- a **nearest-neighbor network** (*47*), where nodes connect to geographically proximate neighbors, producing high local clustering.

Both models preserve the number of nodes, total links, and the share of river connections, ensuring comparability with empirical networks. We generate synthetic networks by reshuffling overland links only, while keeping river connections fixed to reflect geographical constraints. For each empirical network, we generate 100 realizations under each model. This number of realizations guaranteed a large enough sample of synthetic networks, capturing a wide enough range of random-driven structures (and, consequently, network efficiency values).

The procedure is as follows:

1. Identify all links in the empirical network corresponding to land connections, L_{land}.
2. For each link in L_{land}:
   a. Select at random one of the two nodes connected by the selected link, node *a*
   b. Select another node *b*, to be connected to *a*. In the **Homogeneus Random network** case, this would be done at random among all nodes in the network. On the contrary, in the **Nearest-Neighbor network** case, b is chosen to be the closest node in geographical terms. As mentioned in the main text, these designs would stress global and local connectivity, respectively.
   c. If *a* and *b* are not already connected, link them and remove the original link. Otherwise, select another *b* node following instructions at step 2.b.
3. Compute the structural metrics under study for the resulting synthetic network, which presents the same number of nodes and links as the empirical one.

Finally, we use the distributions of structural metrics obtained from those synthetic networks to evaluate the empirical values of each network snapshot. 24 distributions of values (3 time slices X 2 network models X 4 structural metrics) were compared against their empirical counterparts. To do it, boxplot representations were applied (see **Fig. 3** and **Fig. S5**).

5.4. Small-world-ness index

To evaluate the presence of small-world structure, we compute the small-world index proposed by Humphries and Gurney (*38*):

$$S = \text{normalized CC} / \text{normalized APL} = (CC\_{emp}/\text{mean}(CC\_{syn}) / (APL_{emp}/\text{mean}(APL\_{syn}))$$

Where CC_{emp} and APL_{emp} correspond to the clustering coefficient and the average path length of the empirical network, and mean(CC_{syn}) and mean(APL_{syn}) are the means of such metrics calculated for random synthetic counterparts. The index compares empirical clustering and path length to those of synthetic networks. Values above 1 indicate small-world structure.

We adopted a modified version of this original setting by using synthetic network generated by our random network model (instead of 'regular' random networks). As shown in Table S2 below, none of the three S values reached the value of 3, but the value corresponding to LC5 gets very close to 1.

6. Historical Context: Stylized fact.

This section summarizes the historical patterns directly relevant to the structural evolution of the networks analyzed in the main text (*6, 13, 39, 48, 107*):

Prehistoric exchanges (10000-3800 BCE). Following the spread of sedentary communities, interaction across the Fertile Crescent intensified through shared cultural practices, domestication processes, and population movements (*108*). During the Chalcolithic period, settlement systems expanded and became increasingly interconnected. This is reflected in the diffusion of Halaf and Ubaid cultural traditions and in the circulation of materials such as eastern Anatolian obsidian (*8*). Large villages in Northern Mesopotamia acted as regional intermediaries in resource acquisition and redistribution, providing a basis for later interregional exchange systems (*62*, *109*).

Incipient Contact phase *(LC3, 3800-3600 BCE)*. In LC3 (Main text: **Fig. 1a**), settlement systems display marked regional variation, with a complex multi-tier structure in southern Mesopotamia and simpler hierarchies elsewhere, alongside dense occupation across northern regions (*15*). Connectivity is organized primarily along the Tigris and Euphrates corridors, forming elongated chains of settlements. Interaction is mediated through a combination of riverine links and small sites located near key crossings and access points to highland resources (*39*). Major northern centers such as Brak and Hamoukar occupy strategic positions within these chains, linking resource-rich hinterlands to developing southern urban centers (*13*, *15*)

Contact period (*LC4, 3600-3300 BCE)*. In LC4 (Main text: **Fig. 1b**), the network expands both geographically and in number of sites. While river-based connectivity remains important, overland links become more prominent, giving rise to regionally clustered structures. Distinct groups of sites emerge around areas such as Susa, the southern alluvium, and the Euphrates Great Bend, forming identifiable modules within the network (*39*). At the same time, new settlements appear near major river crossings, and several key sites in northern Mesopotamia become incorporated into broader interaction systems. These developments correspond to a shift toward more regionally structured connectivity and increased interregional interaction, attested by the wider distribution of Uruk-related material culture (*5*).

Peak interaction *(LC5, 3300-3000 BCE)*. In LC5 (Main text: **Fig. 1c**), the network consolidates into a smaller number of larger regional clusters, notably centered on Susa, Uruk, and the Euphrates Great Bend (*9*, *39*). Connectivity combines dense local interactions within these clusters with extended chains of settlements along peripheral overland routes. This configuration reflects a balance between localized cohesion and longer-distance connections. At the same time, several large sites emerge at key transit locations, particularly along the Euphrates corridor, which becomes a central axis of interaction (*39*, *54*, *110*). After ca. 3200 BCE, the system undergoes fragmentation, with the abandonment of major northern centers and a decline in overall connectivity (*20*).

FIGURES S1 to S6

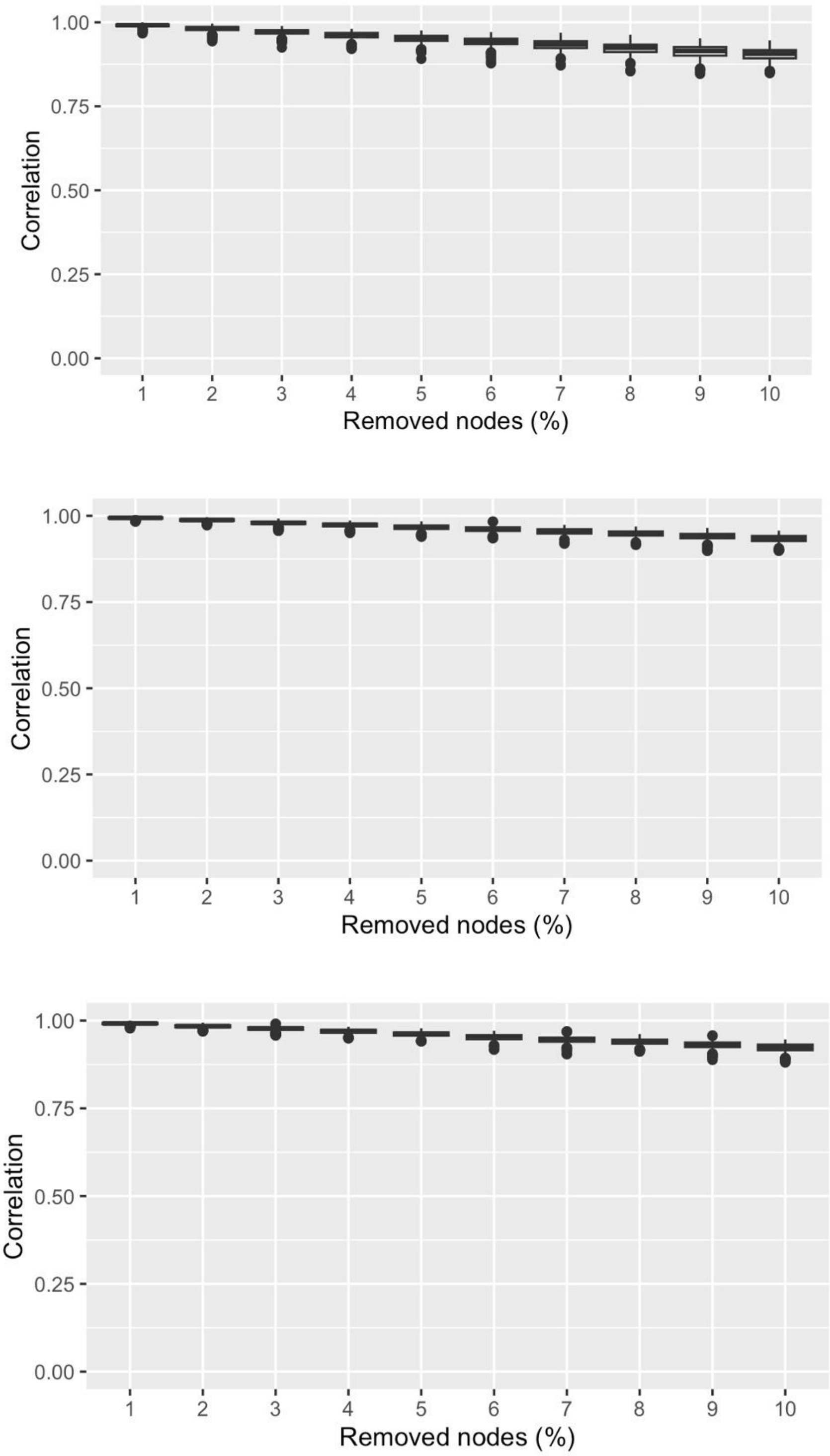

Fig. S1. **Node-removal robustness (degree).** Top: LC3. Middle: LC4. Bottom: LC5. Degree-rank correlations after progressive node removal.

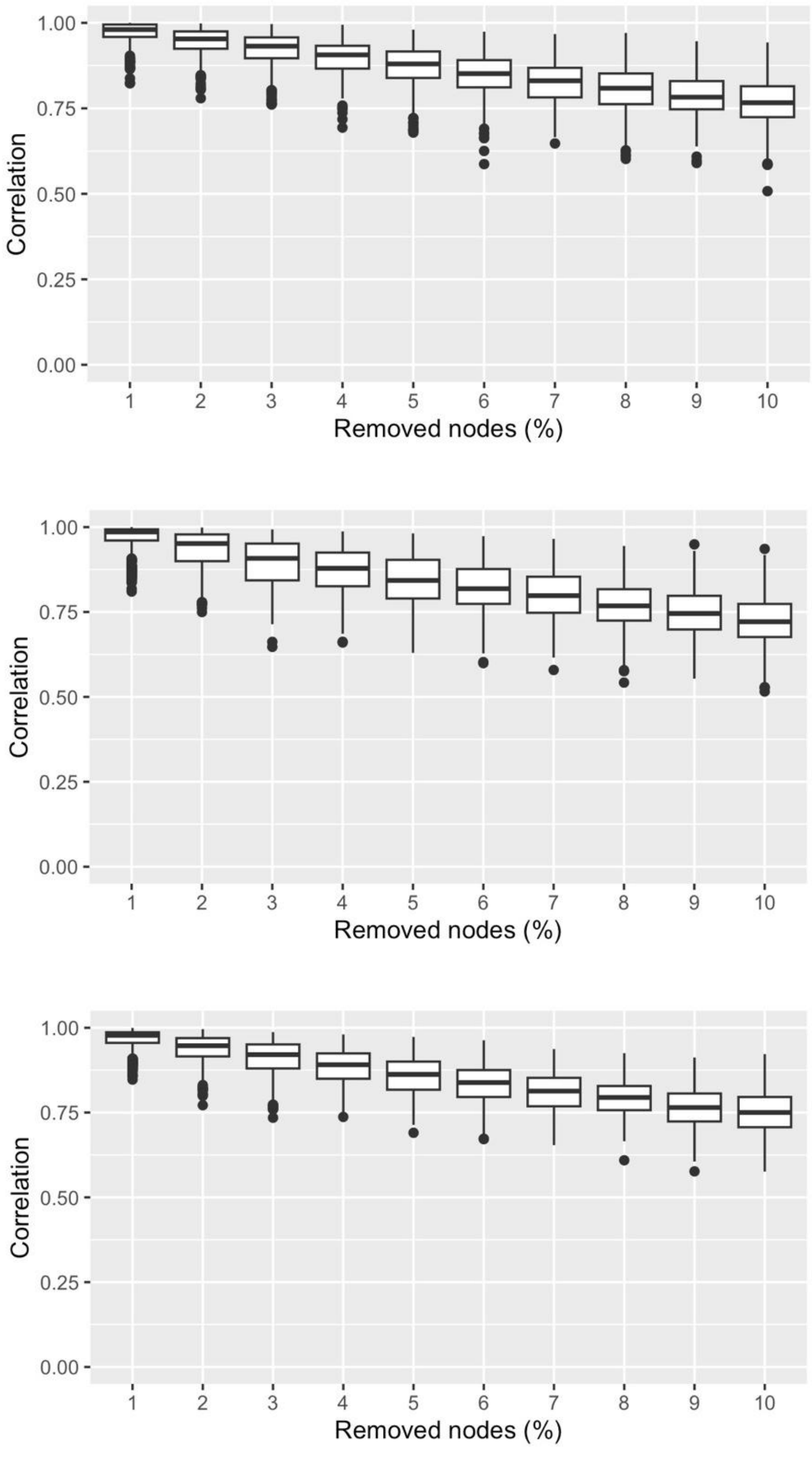

Fig. S2. Node-removal robustness (betweenness). Top: LC3. Middle: LC4. Bottom: LC5. Betweenness-rank correlations after progressive node removal.

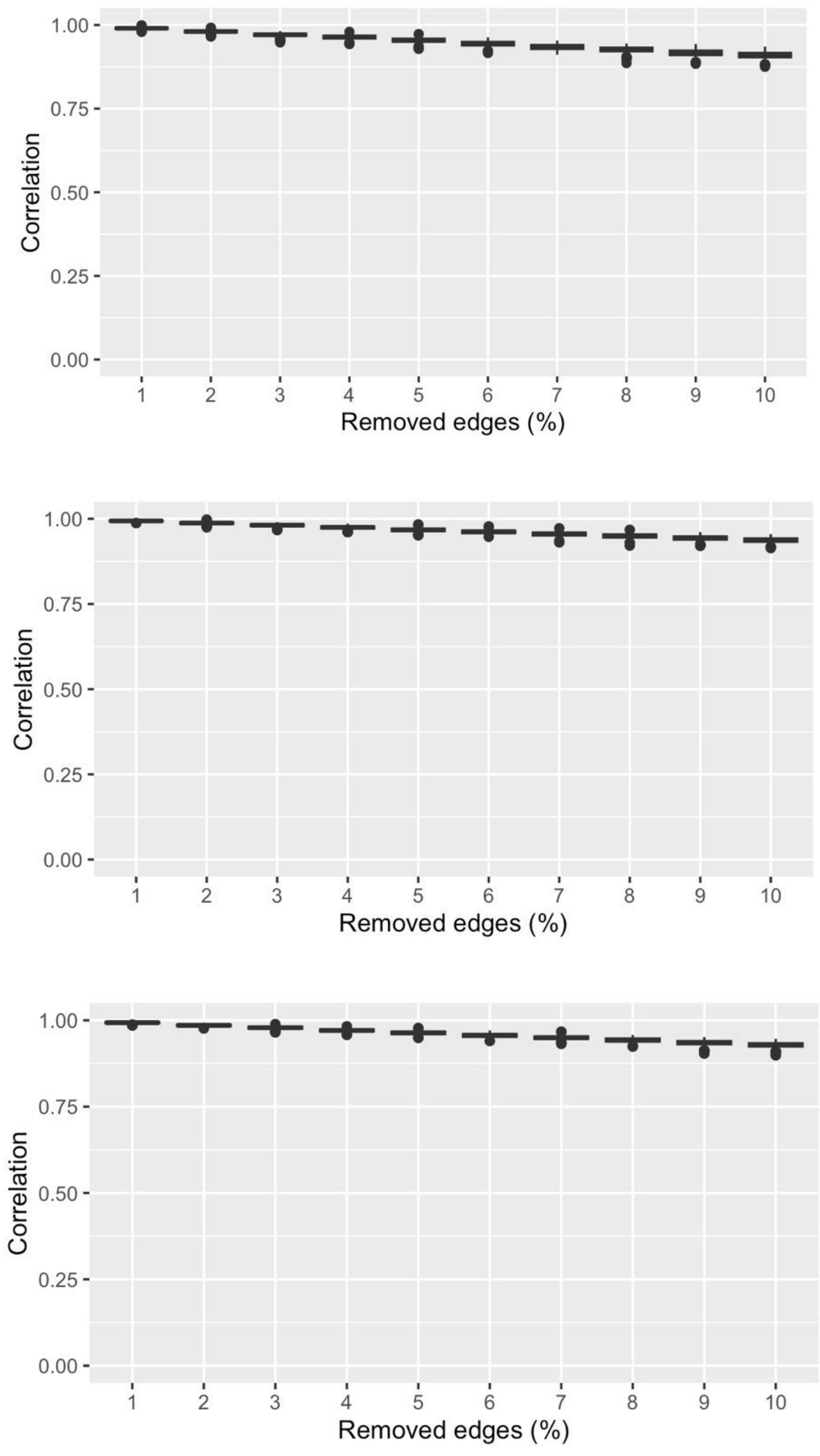

Fig. S3. **Edge-removal robustness (degree).** Top: LC3. Middle: LC4. Bottom: LC5.

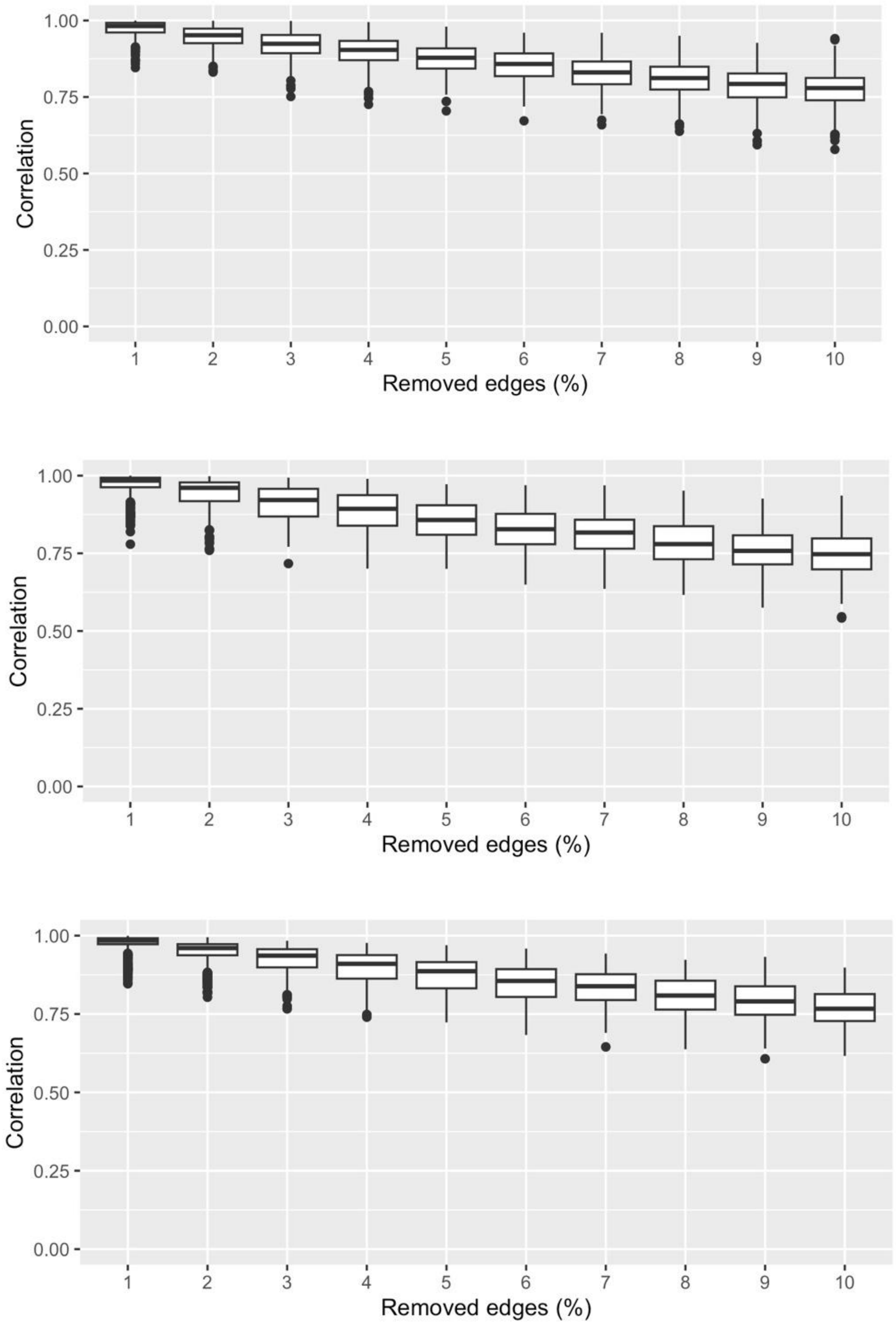

Fig. S4. **Edge-removal robustness (betweenness).** Top: LC3. Middle: LC4. Bottom: LC5.

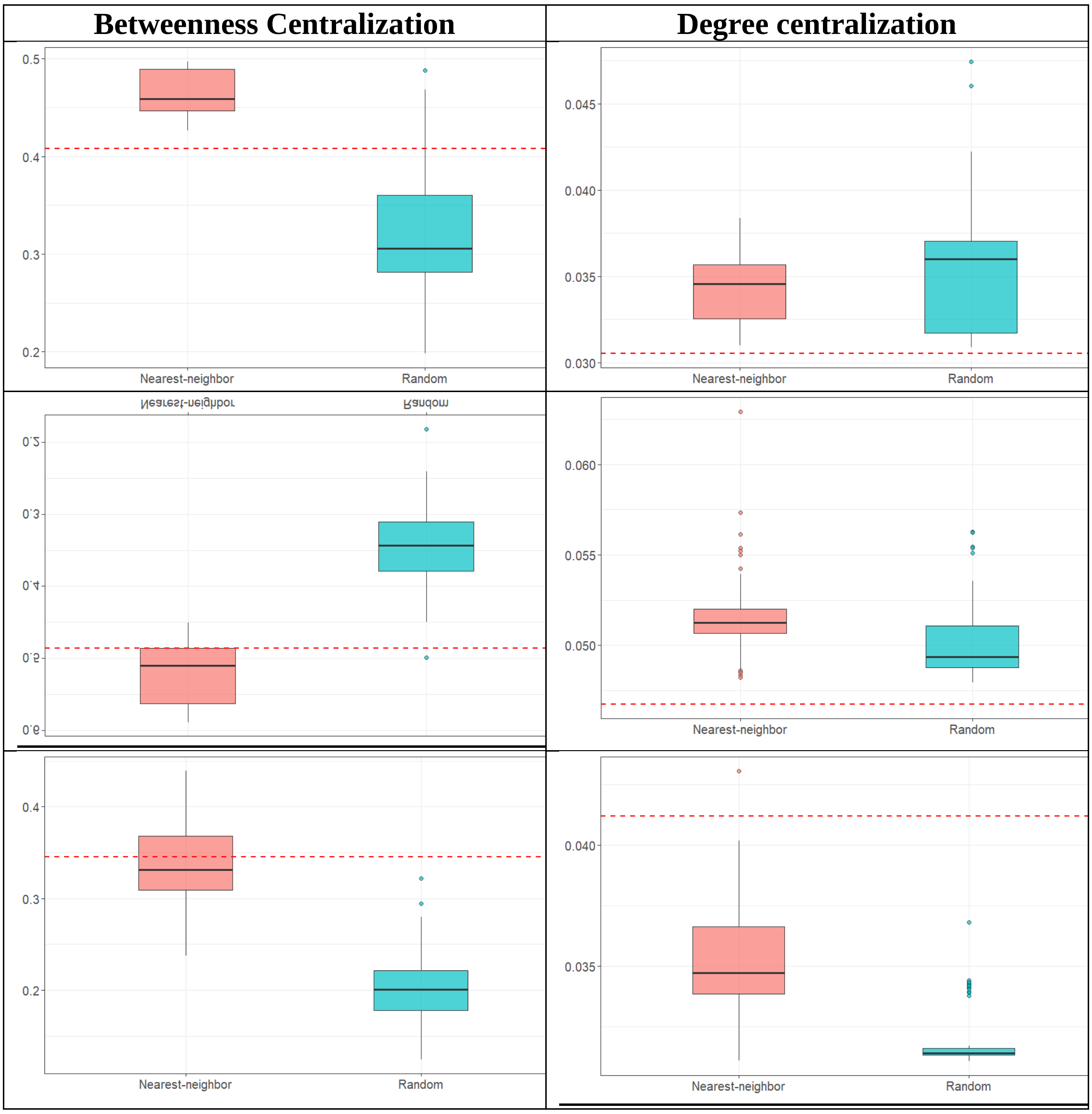

Fig. S5. Computational experiments. Comparison of empirical and synthetic betweenness and degree centralizations (left and right columns, respectively). Results are sorted chronologically from LC3 (top) to LC5 (bottom).

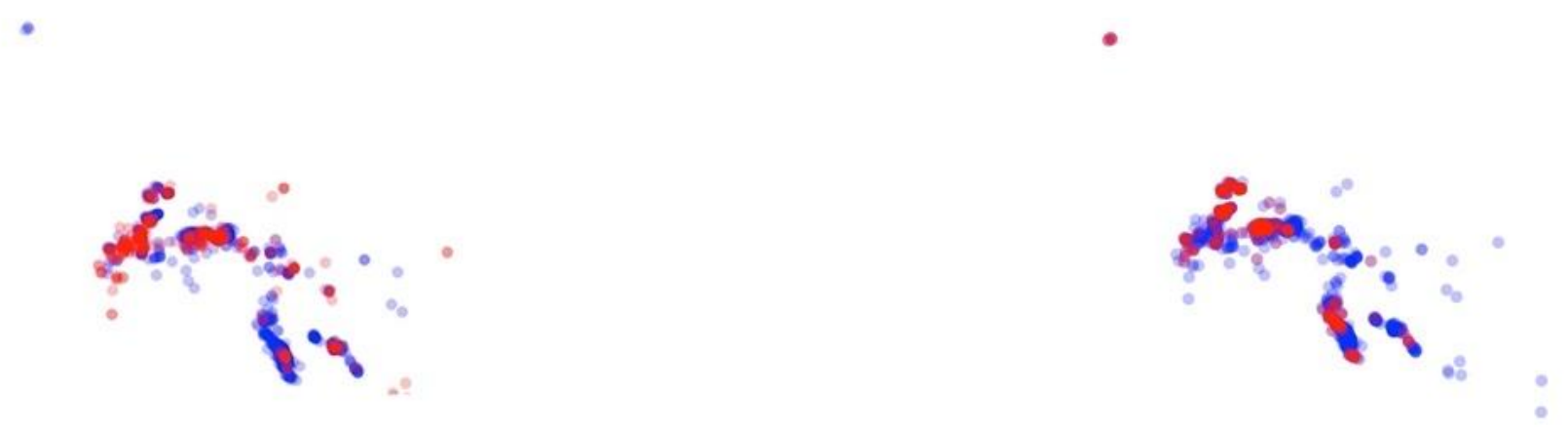

Fig. S6. Including Non-Uruk sites. Comparison of the spatial distribution of regular Uruk sites (in blue) with ‘Non-Uruk’ ones (in red, left side) and Uruk sides without precise-enough chronological information (in red, right side).

TABLES

	Observations	Nodes	
		**Curated**	**Excluded**
LC2-3	410	230	1
LC4	440	335	5
LC5	482	392	6
**Undated/unlocated**	416		84
**Totals**	1748	957	96

Table S1. Curation results for the settlement dataset. Total observations, curated nodes, and excluded nodes by LC phase, as reported in the above Supplementary Text.

	S
LC3	0.85
LC4	0.80
LC5	0.99

Table S2. Values of small-world-ness. Index S calculated for the three empirical networks. See section 5.4 in the Supplementary Text.

Data S1 (separate file).

Site-level metadata (coordinates, chronology, Uruk cultural markers, references).

Data S2 (separate file).

R code for network construction, robustness tests, synthetic network generation, and figure/table reproduction.

REFERENCES

1. K. Kristiansen, T. Lindkvist, J. Myrdal, *Trade and Civilisation. Economic Networks and Cultural Ties, From Prehistory to the Early Modern Era* (Cambridge Univ. Press, 2018).

2. M. Liverani, *The Ancient Near East. History, Society, Economy* (Routledge, 2014).

3. J. Jennings, *Globalizations and the Ancient World* (Cambridge Univ. Press, 2011).

4. M. Massa, A. Palmisano, Change and continuity in the long-distance exchange networks between Western/Central Anatolia, Northern Levant and Northern Mesopotamia, c. 3200–1600 BCE. *J. Anthropol. Archaeol.* **49**, 65–87 (2018).

5. L. D. Minc, G. Emberling, Trade and interaction during the era of the Uruk expansion: Recent insights from archaeometric analyses. *J. Archaeol. Sci. Rep.* **7**, 793–797 (2016).

6. G. Algaze, "The end of prehistory and the Uruk period," in *The Sumerian World*, H. Crawford, Ed. (Routledge, 2013), pp. 68–94.

7. J. J. Ibáñez, D. Ortega, D. Campos, L. Khalidi, V. Mendez, L. Teira, Developing a complex network model of obsidian exchange in the neolithic near east: Linear regressions. ethnographic models and archaeological data. *Paléorient* **42**, 9–32 (2016).

8. L. Khalidi, B. Gratuze, G. J. Stein, A. McMahon, S. Al-Quntar, R. Carter, R. Cuttler, P. Drechsler, E. Healey, M.-L. Inizan, D. Mouralis, E. Pernicka, A.-K. Robin, The Growth of early social networks: New geochemical results of obsidian from the Ubaid to Chalcolithic Period in Syria, Iraq and the Gulf. *J. Archaeol. Sci. Rep.* **9**, 743–757 (2016).

9. G. Algaze, *Ancient Mesopotamia at the Dawn of Civilization. The Evolution of an Urban Landscape* (University of Chicago Press, 2008).

10. G. Algaze, *The Uruk World System. The Dynamics of Expansion of Early Mesopotamian Civilization* (Chicago Univ. Press, 1993).

11. C. Chase-Dunn, E. N. Anderson, *The Historical Evolution of World-Systems* (Palgrave Macmillan, 2005).

12. G. Algaze, "The prehistory of imperialism: The case of Uruk Period Mesopotamia," in *Uruk Mesopotamia & Its Neighbors. Cross Cultural Interactions in the Era of State Formation*, M. S. Rothman, Ed. (SAR Press, 2001), pp. 27–83.

13. G. J. Stein, The development of indigenous social complexity in Late Chalcolithic Upper Mesopotamia in the 5th-4th Millennia BC—An initial assessment. *Origini* **24**, 115–142 (2012).

14. M. Frangipane, Different trajectories in state formation in Greater Mesopotamia: A view from Arslantepe (Turkey). *J. Archaeol. Res.* **26**, 3–63 (2018).

15. A. McMahon, Early urbanism in Northern Mesopotamia. *J. Archaeol. Res.* **28**, 289–337 (2020).

16. G. J. Stein, *Rethinking World-Systems. Diasporas, Colonies and Interaction in Uruk Mesopotamia* (University of Arizona Press, 1999).

17. M. S. Rothman, *Uruk Mesopotamia & Its Neighbors. Cross-Cultural Interaction in the Era of State Formation* (School of American Research Press, 2001).

18. G. Algaze, Entropic cities: The paradox of urbanism in Ancient Mesopotamia. *Curr. Anthropol.* **59**, 23–54 (2018).

19. A. B. Porter, *Mobile Pastoralism and the Formation of Near Eastern Civilizations: Weaving Together Society* (Cambridge Univ. Press, 2012).

20. H. J. Nissen, "Cultural and political networks in the ancient near East during the fourth and third millennia B.C.," in *Uruk Mesopotamia & Its Neighbors. Cross Cultural Interactions in the Era of State Formation*, M. Rothman, Ed. (School of American Research Press, 2001), pp. 149–180.

21. G. A. Johnson, Late Uruk in Greater Mesopotamia: Expansion or collapse? *Origini* **14**, 595–613 (1989).

22. M. Flückiger, M. Larch, M. Ludwig, L. Pascali, The dawn of civilization metal trade and the rise of hierarchy (American Economic Association, 2024); https://doi.org/10.2139/ssrn.4723722.

23. T. Brughmans, M. A. Peeples, *Network Science in Archaeology* (Cambridge Univ. Press, 2023).

24. T. Brughmans, B. J. Mills, J. Munson, M. A. Peeples, *The Oxford Handbook of Archaeological Network Research* (Oxford Univ. Press, 2023).

25. R. L. Kelly, M. E. Mackie, A. W. Kandel, Rapid increase in production of symbolic artifacts after 45,000 years ago is not a consequence of taphonomic bias. *J. Archaeol. Sci.* **160**, 105885 (2023).

26. M. Altaweel, A. Palmisano, Urban and transport scaling: Northern Mesopotamia in the Late Chalcolithic and Bronze Age. *J. Archaeol. Method Theory* **26**, 943–966 (2019).

27. D. Lawrence, A. Palmisano, M. W. de Gruchy, Collapse and continuity: A multi-proxy reconstruction of settlement organization and population trajectories in the Northern Fertile Crescent during the 4.2kya Rapid Climate Change event. *PLOS ONE* **16**, e0244871 (2021).

28. N. Marchetti, E. Bortolini, J. C. Menghi Sartorio, V. Orrù, F. Zaina, Long-term urban and population trends in the Southern Mesopotamian floodplains. *J. Archaeol. Res.* **33**, 117–158 (2024).

29. D. Priß, J. Wainwright, D. Lawrence, L. Turnbull, C. Prell, C. Karittevlis, A. A. Ioannides, Filling the gaps—Computational approaches to incomplete archaeological networks. *J. Archaeol. Method Theory* **32**, 19 (2025).

30. M. L. Smith, Networks, territories, and the cartography of ancient states. *Ann. Assoc. Am. Geogr.* **95**, 832–849 (2005).

31. M. R. Groenhuijzen, P. Verhagen, Testing the robustness of local network metrics in research on archeological local transport networks. *Front. Digit. Human.* **3**, 00006 (2016).

32. M. Matteazzi, All the roads to Patavium: Morphology, genesis and development of the roman road network around Padua. *Open Archaeol.* **3**, 83–100 (2017).

33. A. Herranz-Loncán, J. Fourie, "For the public benefit"? Railways in the British Cape Colony. *Eur. Rev. Econ. Hist.* **22**, 73–100 (2018).

34. L. Prignano, I. Morer, F. Fulminante, S. Lozano, Modelling terrestrial route networks to understand inter-polity interactions (Southern Etruria, 950-500 BC). *J. Archaeol. Sci.* **105**, 46–58 (2019).

35. F. Pablo-Martí, Á. Alañón-Pardo, A. Sánchez, Complex networks to understand the past: the case of roads in Bourbon Spain. *Cliometrica* **15**, 477–534 (2021).

36. N. D. Conrad, R. Chemnitz, M. Kostré, F. Schweigart, F. Fless, C. Schütte, B. Ducke, A mathematical perspective on Romanisation: Modelling the Roman road activation process in ancient Tunisia. *PLOS ONE* **19**, e0309752 (2024).

37. V. D. Blondel, J. L. Guillaume, R. Lambiotte, E. Lefebvre, Fast unfolding of communities in large networks. *J. Stat. Mech*, 10008–10012 (2008).

38. M. E. J. Newman, Models of the small world. *J. Stat. Phys.* **101**, 819–841 (2000).

39. G. Algaze, "The transregional origins of Early Mesopotamian civilization," in *The 'City' across Time. Emergence, Developments, and Social, Economic Political, Cultural and Health Impact*, M. Frangipane, Ed. (Bardi Edizioni, 2023).

40. M. D. Humphries, K. Gurney, Network 'small-world-ness': A quantitative method for determining canonical network equivalence. *PLOS ONE* **3**, e0002051 (2008).

41. K. V. Flannery, J. Marcus, *The Creation of Inequality: How Our Prehistoric Ancestors Set the Stage for Monarchy, Slavery and Empire* (Harvard Univ. Press, 2012).

42. L. D. Minc, Monitoring regional market systems in prehistory: Models, methods, and metrics. *J. Anthropol. Archaeol.* **25**, 82–116 (2006).

43. B. Bollobas, *Random Graphs* (Cambridge Univ. Press, 2001).

44. A. K. Styring, M. P. Charles, F. Fantone, M. M. Hald, A. McMahon, R. Meadow, G. K. Nicholls, A. K. Patel, M. C. Pitre, A. Smith, A. Sołtysiak, G. J. Stein, J. Weber, H. Weiss, A. Bogaard, Isotope evidence for agricultural extensification reveals how the world's first cities were fed. *Nat. Plants* **3**, 17076 (2017).

45. G. J. Stein, From passive periphery to active agents: Emerging Perspectives in the Archaeology of Interregional Interaction: Archeology Division Distinguished Lecture AAA Annual Meeting, Philadelphia, December 5, 1998. *Am. Anthropol.* **104**, 903–916 (2002).

46. M. Á. Serrano, M. Boguñá, Topology of the world trade web. *Phys. Rev. E* **68**, 015101 (2003).

47. J. Dall, M. Christensen, Random geometric graphs. *Phys. Rev. E* **66**, 016121 (2002).

48. P. Butterlin, *Les Bâtisseurs de Mémoire En Mésopotamie (7000–3000 Av. J.-C.)* (Picard, 2018).

49. G. M. Feinman, C. P. Garraty, Preindustrial markets and marketing: Archaeological perspectives. *Ann. Rev. Anthropol.* **39**, 167–191 (2010).

50. R. C. Oka, C. M. Kusimba, The archaeology of trading systems, part 1: Towards a new trade synthesis. *J. Archaeol. Res.* **16**, 339–395 (2008).

51. A. Ossa, Using network expectations to identify multiple exchange systems: A case study from Postclassic Sauce and its hinterland in Veracruz, Mexico. *J. Anthropol. Archaeol.* **32**, 415–432 (2013).

52. H. T. Wright, The Uruk expansion and beyond: Archaeometric and social perspectives on exchange in the IVth millennium BCE. *J. Archaeol. Sci. Rep.* **7**, 900–904 (2016).

53. G. J. Stein, "Colonies without Colonialism: A Trade Diaspora Model of Fourth Millennium BC Mesopotamian Enclaves in Anatolia," in *The Archaeology of Colonialism*, C. Lyons, J. Papadopoulos, Eds. (Getty Research Institute, 2002), pp. 27–64.

54. L. Blaydes, C. Paik, Old world trade diasporas. *Sociol. Sci.* **11**, 1–41 (2024).

55. C. A. Petrie, F. K. Jürcke, T. C. Wilkinson, H. A. Orengo, Over the hills and far away: Modeling mobility and connectivity across the Iranian Plateau in Late Prehistory (c. 10,000–2000 BC) using multi-factor probabilistic corridors. *J. Archaeol. Res.* **34**, 557–614 (2026).

56. H. Pittman, M. J. Blackman, Mobile or stationary? Chemical analysis of clay administrative devices from Tell Brak in the Uruk Period. *J. Archaeol. Sci. Rep.* **7**, 877–883 (2016).

57. L. D. Minc, Style and substance: Evidence for regionalism within the Aztec market system. *Lat. Am. Antiq.* **20**, 343–374 (2009).

58. S. C. Clayton, Contextualizing commerce at Teotihuacan: Pottery as evidence for regional and neighborhood-scale markets. *Archaeol. Pap. Am. Anthropol. Assoc.* **32**, 43–53 (2021).

59. J. K. Millhauser, L. Overholtzer, Commodity chains in archaeological research: Cotton cloth in the Aztec economy. *J. Archaeol. Res.* **28**, 187–240 (2020).

60. G. M. Feinman, L. M. Nicholas, M. Golitko, "Macroscale shifts in Obsidian procurement networks across prehispanic Mesoamerica," in *Obsidian Across the Americas*, G. M. Feinman, D. J. Riebe, Eds. (Archaeopress Publishing Ltd, 2022), pp. 98–123.

61. C. D. Dillian, C. A. Bello, M. S. Shackley, "Long-distance exchange of obsidian in the mid-Atlantic United States," in *Trade and Exchange*, C. D. Dillian, C. L. White, Eds. (Springer New York, 2010), pp. 17–35.

62. O. Barge, C. Chataigner, Diffusion of Anatolian and Caucasian obsidian in the Zagros Mountains and the highlands of Iran: Elements of explanation in 'least cost path' models. *Quat. Int.* **467**, 297–322 (2018).

63. F. Fulminante, *The Rise of Early Rome: Transportation Networks and Domination in Central Italy, 1050–500 BC* (Cambridge Univ. Press, 2023).

64. A. W. Marrs, *Railroads in the Old South: Pursuing Progress in a Slave Society* (Johns Hopkins Univ. Press, 2009).

65. D. Donaldson, R. Hornbeck, Railroads and American Economic Growth: A "Market Access" Approach. *Q. J. Econ.* **131**, 799–858 (2016).

66. A. Wilhite, Bilateral trade and 'small-world' networks. *Comput. Econ.* **18**, 49–64 (2001).

67. J. Pournelle, "Physical Geography," in *The Sumerian World*, H. Crawford, Ed. (Routledge, 2013), pp. 13–32.

68. W. B. F. Ryan, S. M. Carbotte, J. O. Coplan, S. O'Hara, A. Melkonian, R. Arko, R. A. Weissel, V. Ferrini, A. Goodwillie, F. Nitsche, J. Bonczkowski, R. Zemsky, Global multi-resolution topography synthesis. *Geochem. Geophys. Geosyst.* **10**, GC002332 (2009).

69. M. Bastian, S. Heymann, M. Jacomy, Gephi: An open source software for exploring and manipulating networks. *Proc. Int. AAAI Conf. Web Soc. Media* **3**, 361–362 (2009).

70. C. Vignola, F. Marzaioli, F. Balossi Restelli, G. M. Di Nocera, M. Frangipane, A. Masi, I. Passariello, L. Sadori, F. Terrasi, Changes in the Near Eastern Chronology Between the 5th and the 3rd millennium BC: New AMS 14C dates from Arslantepe (Turkey). *Nucl. Instrum. Methods Phys. Res. B* **456**, 276–282 (2019).

71. M. van Ess, K.-U. Heußner, Absolute Chronology of the Uruk and Jemdet Nasr Periods at Uruk, Southern Mesopotamia. The Interpretation of Additional 14C Samples. *Z. für Orient-Archäol.* **8**, 10–37 (2015).

72. M. M. Wencel, Towards an absolute chronology of early Mesopotamia: A radiocarbon perspective, thesis, University of Oxford (2018).

73. J. Dahl, C. Petrie, D. T. Potts, "Chronological parameters of the earliest writing system in Iran," in *Ancient Iran and Its Neighbours. Local Developments and Long-Range Interactions in the Fourth Milenium BC (British Institute of Persian Studies. Archaeological Monographs Series, III)*, C. Petrie, Ed. (Oxbow Books, 2013), pp. 353–378.

74. S. Renette, M. P. Lewis, M. M. Wencel, A. Farahani, A. Tomé, Establishing an absolute chronological framework for the Late Chalcolithic to Early Bronze Age in Iraqi Kurdistan: Rardiocarbon dates from Kani Shaie. *Radiocarbon* **65**, 209–231 (2023).

75. P. Butterlin, L"Expansion de la Culture D'uruk, Uruk et les Colonies urukéennes, à la Fin du IV e millénaire in *Atlas Historique Du Proche-Orient Ancien*, M. Sauvage, Ed. (Les Belles Lettres, Ifpo, Paris - Beyrouth, 2020), pp. 36–38.

76. O. Pedersen, ANE Site Placemarks for Google Earth (Version 10) (Zenodo, 2007); https://doi.org/https://doi.org/10.5281/zenodo.6384045.

77. D. Lawrence, G. J. Philip, H. Hunt, L. Snape-Kennedy, T. J. Wilkinson, Long term population, city size and climate trends in the fertile crescent: A first approximation. *PLOS ONE* **11**, e0157863 (2016).

78. A. Palmisano, D. Lawrence, M. W. de Gruchy, A. Bevan, S. Shennan, Holocene regional population dynamics and climatic trends in the Near East: A first comparison using archaeo-demographic proxies. *Quat. Sci. Rev.* **252**, 106739 (2021).

79. K. Sundsal, The Uruk expansion: Culture contact, ideology and middlemen. *Nor. Archaeol. Rev.* **44**, 164–185 (2011).

80. G. Algaze, The Uruk expansion: Cross-cultural exchange in Early Mesopotamian Civilization. *Curr. Anthropol.* **30**, 571–608 (1989).

81. H. T. Wright, "Cultural action in the Uruk World," in *Uruk Mesopotamia and Its Neighbors*, M. Rothmann, Ed. (SAR Press, 2001), pp. 123–148.

82. H. Pittman, "Imagery in Administrative Context: Susiana and the West in the Fourth Millennium BC," in *Ancient Iran and Its Neighbours. Local Developments and Long-Range Interactions in the Fourth Milenium BC (British Institute of Persian Studies. Archaeological Monographs Series, III)*, C. A. Petrie, Ed. (British Institute of Persian Studies/Oxbow Books, 2013), pp. 293–336.

83. P. Butterlin, "Late Chalcolithic Mesopotamia: Towards a Definition of Sacred Space and Its Evolution," in *Defining the Sacred. Approaches to the Archaeology of Religion in the Near East*, N. Laneri, Ed. (Oxbow Books, Oxford, 2015), pp. 60–72.

84. B. H. Menze, J. Ur, Mapping patterns of long-term settlement in Northern Mesopotamia at a large scale. *Proc. Natl. Acad. Sci. U.S.A.* **109**, E778–E787 (2012).

85. T. C. Wilkinson, *Tying the Threads of Eurasia. Trans-Regional Routes and Material Flows in Transcaucasia, Eastern Anatolia and Western Central Asia, c. 3000-1500BC* (Sidestone Press, 2014).

86. G. Barjamovic, "Interlocking commercial networks and the infrastructure of trade in Western Asia during the Bronze Age," in *Trade and Civilisation: Economic Networks and Cultural Ties from Prehistory to the Early Modern Era*, K. Kristiansen, T. Lindkvist, J. Myrdal, Eds. (Cambridge Univ. Press, 2018), pp. 113–142.

87. A. Palmisano, *The Geography of Trade Landscapes of Competition and Long-Distance Contacts in Mesopotamia and Anatolia in the Old Assyrian Colony Period* (Archaeopress, Oxford, 2018).

88. C. Marro, "Upper Mesopotamia and the Caucasus: An essay on the Evolution of Routes and Road Networks from the Old Assyrian Kingdom to the Ottoman Empire," in *A View from the Highlands: Archaeological Studies in Honour of Charles Burney*, A. Sagona, Ed. (Peeters, 2004), pp. 91–120.

89. G. Barjamovic, T. Chaney, K. Coşar, A. Hortaçsu, Trade, merchants, and the lost cities of the Bronze Age. *Q. J. Econ.* **134**, 1455–1503 (2019).

90. M. J. Stahlberg, G. Sagnol, B. Ducke, M. Klimm, Spatiotemporal reconstruction of ancient road networks through sequential cost–benefit analysis. *PNAS Nexus* **2**, 313 (2023).

91. R. J. Matthews, H. Fazeli, *The Archaeology of Iran from the Palaeolithic to the Achaemenid Empire* (Routledge, 2022).

92. T. J. Wilkinson, N. F. Miller, C. D. Reichel, D. Whitcomb, *On the Margin of the Euphrates: Settlement and Land Use at Tell Es-Sweyhat and in the Upper Lake Assad Area, Syria* (The Oriental Institute, 2004).

93. M. W. de Gruchy, Routes of the Uruk Expansion, thesis, Durham University (2017).

94. J. Jotheri, Holocene avulsion history of the Euphrates and Tigris rivers in the Mesopotamian floodplain," thesis, Durham University (2016).

95. T. J. Wilkinson, C. Hritz, "Physical geography, environmental change and the role of water," in *Models of Mesopotamian Landscapes. How Small-Scale Processes Contributed to the Growth of Early Civilizations*, T. J. Wilkinson, M. Gibson, M. Widell, Eds. (Archaeopress, 2013), pp. 9–33.

96. S. Rost, Water management in Mesopotamia from the sixth till the first millennium B.C. *Wiley Interdiscip. Rev. Water* **4**, 1–23 (2017).

97. R. C. Allen, M. C. Bertazzini, L. Heldring, The economic origins of government. *Am. Econ. Rev.* **113**, 2507–2545 (2023).

98. R. M. Adams, *Heartland of Cities. Surveys of Ancient Settlement and Land Use on the Central Floodplain of the Euphrates* (University of Chicago Press, 1981).

99. J. Pournelle, Marshland of Cities: Deltaic Landscapes and the Evolution of Early Mesopotamian Civilization, thesis, Universtiy of California, San Diego (2003).

100. J. Pournelle, "The Littoral Foundations of the Uruk State: Using satellite photography toward a new understanding of 5th/4th millennium BCE landscapes in the Warka Survey Area, Iraq," in *Chalcolithic and Early Bronze Age Hydrostrategies*, D. Gheorghiou, Ed. (Archaeopress, 2003), pp. 5–24.

101. J. Pournelle, "From KLM to Corona: Using Satellite Photography toward a New Understanding of the 5th/4th Millennium BC Landscapes in Southern Mesopotamia," in *Settlement and Society. Essays Dedicated to Robert McCormick Adams (Ideas, Debates, and Perspectives Vol. 3)*, E. C. Stone, Ed. (Cotsen Institute of Archaeology and the Oriental Institute of the University of Chicago, 2007), pp. 29–62.

102. R. M. Adams, *The Land Behind Baghdad* (University of Chicago Press, 1965).

103. G. A. Johnson, *Local Exchange and Early State Development in Southwestern Iran (Anthropological Papers, University of Michigan Museum of Anthropology, 51)* (University of Michigan, 1973).

104. A. Alizadeh, *Ancient Settlement Systems and Culture in the Ram Hormuz Plain, Southwestern Iran. Excavations at Tall e-Geser and Regional Survey of the Ram Hormuz Area (OIP 140)* (The Oriental Institute of the University of Chicago, 2014).

105. D. J. Kennett, J. P. Kennett, "Influence of holocene marine transgression and climate change on cultural evolution," in Southern Mesopotamia in *Climate Change & Cultural Dynamics. A Global Perspective on Mid-Holocene Transitions*, D. G. Anderson, K. A. Maasch, D. H. Sandweiss, Eds. (Academic Press, 2007), pp. 229–264.

106. E. R. Crema, Statistical modelling in archaeology: Some recent trends and future perspectives. *J. Archaeol. Sci.* **180**, 106295 (2025).

107. G. Algaze, Initial social complexity in Southwestern Asia: The Mesopotamian advantage. *Curr. Anthropol.* **42**, 199–233 (2001).

108. J. J. Ibáñez, D. Ortega, D. Campos, L. Khalidi, V. Méndez, Testing complex networks of interaction at the onset of the Near Eastern Neolithic using modelling of obsidian exchange. *J. R. Soc. Interface* **12**, 20150210 (2015).

109. M. Schwartz, D. Hollander, The Uruk expansion as dynamic process: A reconstruction of middle to Late Uruk exchange patterns from bulk stable isotope analyses of Bitumen artifacts. *J. Archaeol. Sci. Rep.* **7**, 884–899 (2016).

110. P. Butterlin, "Princes Marchands D'uruk? L'expansion urukéenne en Question, études Proto-Urbaines 5," in *Urbanized Landscapes in Early Syro-Mesopotamia and Prehispanic Mesoamerica Papers of a Cross-Cultural Seminar Held in Honor of Robert McCormick Adams*, D. Domenici, N. Marchetti, Eds. (Harrassowitz, 2020), pp. 71–102.
